# Burning Fog: Cognitive Impairment in Burning Mouth Syndrome

**DOI:** 10.3389/fnagi.2021.727417

**Published:** 2021-08-12

**Authors:** Federica Canfora, Elena Calabria, Renato Cuocolo, Lorenzo Ugga, Giuseppe Buono, Gaetano Marenzi, Roberta Gasparro, Giuseppe Pecoraro, Massimo Aria, Luca D'Aniello, Michele Davide Mignogna, Daniela Adamo

**Affiliations:** ^1^Department of Neurosciences, Reproductive Sciences and Dentistry, University of Naples “Federico II”, Naples, Italy; ^2^Department of Clinical Medicine and Surgery, University of Naples “Federico II”, Naples, Italy; ^3^Department of Advanced Biomedical Sciences, University of Naples “Federico II”, Naples, Italy; ^4^Department of Diagnostical Morphological and Functional, University of Naples “Federico II”, Naples, Italy; ^5^Department of Economics and Statistics, University of Naples “Federico II”, Naples, Italy; ^6^Department of Economics and Statistics, University of Campania “Luigi Vanvitelli”, Caserta, Italy

**Keywords:** mild cognitive impairment, burning mouth syndrome, mini mental, trail making, mood disorders

## Abstract

**Background:** Due to its common association with chronic pain experience, cognitive impairment (CI) has never been evaluated in patients with burning mouth syndrome (BMS). The purpose of this study is to assess the prevalence of CI in patients with BMS and to evaluate its relationship with potential predictors such as pain, mood disorders, blood biomarkers, and white matter changes (WMCs).

**Methods:** A case-control study was conducted by enrolling 40 patients with BMS and an equal number of healthy controls matched for age, gender, and education. Neurocognitive assessment [Mini Mental State Examination (MMSE), Digit Cancellation Test (DCT), the Forward and Backward Digit Span task (FDS and BDS), Corsi Block-Tapping Test (CB-TT), Rey Auditory Verbal Learning Test (RAVLT), Copying Geometric Drawings (CGD), Frontal Assessment Battery (FAB), and Trail Making A and B (TMT-A and TMT-B)], psychological assessment [Hamilton Rating Scale for Depression and Anxiety (HAM-D and HAM-A), Pittsburgh Sleep Quality Index (PSQI), Epworth Sleepiness Scale (ESS), and 36-Item Short Form Health Survey (SF-36)], and pain assessment [Visual Analogic Scale (VAS), Total Pain Rating index (T-PRI), Brief Pain Inventory (BPI), and Pain DETECT Questionnaire (PD-Q)] were performed. In addition, blood biomarkers and MRI of the brain were recorded for the detection of Age-Related WMCs (ARWMCs). Descriptive statistics, the Mann-Whitney *U*-test, the Pearson Chi-Squared test and Spearman's correlation analysis were used.

**Results:** Patients with BMS had impairments in most cognitive domains compared with controls (*p* < 0.001^**^) except in RAVLT and CGD. The HAM-D, HAM-A, PSQI, ESS, SF-36, VAS, T-PRI, BPI and PD-Q scores were statistically different between BMS patients and controls (*p* < 0.001^**^) the WMCs frequency and ARWMC scores in the right temporal (RT) and left temporal (LT) lobe were higher in patients with BMS (*p* = 0.023^*^).

**Conclusions:** Meanwhile, BMS is associated with a higher decline in cognitive functions, particularly attention, working memory, and executive functions, but other functions such as praxis-constructive skills and verbal memory are preserved. The early identification of CI and associated factors may help clinicians to identify patients at risk of developing time-based neurodegenerative disorders, such as Alzheimer's disease (AD) and vascular dementia (VD), for planning the early, comprehensive, and multidisciplinary assessment and treatment.

## Introduction

The number of older people is rising, and the global prevalence of individuals aged more than 60 years will approximately double from about 12 to 22% by 2050 (Christensen et al., 2009). Due to this aging population, dementia (currently 50 million diagnosed cases), chronic pain (30.8%), and depression (7%) represent the prevalence of serious comorbidities in the elderly population, which cause a great impact on the economy, with the estimation of the annual global cost to be over 1 trillion USD (Zis et al., 2017).

For this reason, the early detection of these conditions is becoming crucial for health-care providers (Zelaya et al., 2020). Recently, it has been reported that chronic pain is associated with the increases in a self-rated and an objective cognitive impairment (CI) (Whitlock et al., 2017). Indeed, in epidemiological studies on community-dwelling residents and pain clinics, it has been estimated that wherever the intensity of pain is positively correlated with the degree of CI, at least 50% of people living with pain showed an impairment in objective cognitive tests (Whitlock et al., 2017; Cao et al., 2019).

Burning mouth syndrome (BMS) is a type of chronic neuropathic oral pain disorder characterized by a generalized or localized burning or dysesthetic sensation of the oral mucosa without the evidence of any specific mucosal lesions and/or laboratory findings [International Classification of Orofacial Pain, 2020]. Oral burning sensations are usually bilateral with fluctuating intensity lasting more than 2 h per day for more than 3 months. Xerostomia, taste disturbance, intraoral foreign body sensation, itching, and tingling sensation (Grushka et al., 2003) have been frequently reported (Grushka et al., 2003; Adamo et al., 2015, 2018, 2020). Eating and drinking may sometimes help to alleviate symptomatology. Compared to men, BMS occurs more often in middle-aged women, especially those who are experiencing menopause with a prevalence of 0.7–4.6% (Khan et al., 2014; Kohorst et al., 2015; Wu et al., 2021).

Its pathogenesis remains debated, but it is reasonable to assume a multifactorial process, including psychological factors, central nervous system dysfunctioning, peripheral small fiber neuropathy (Yilmaz et al., 2007; Cazzato and Lauria, 2017), and inflammatory factors (Barry et al., 2018; Pereira et al., 2020). Neuroimaging has provided the evidence of structural and functional brain changes in BMS and in other neuropathic chronic pain (NCP) conditions, which are also commonly reported in neurodegenerative diseases such as Alzheimer's disease (AD) (Tariq et al., 2018) and vascular dementia (VD) (Martucci et al., 2014; Tu et al., 2020). Recent studies have proposed a correlation between NCP and cognitive decline, suggesting the presence of a bidirectional interaction (Povedano et al., 2007; Femir-Gurtuna et al., 2020). Indeed, untreated pain could accelerate the onset of neurodegenerative diseases, and at the same time, a cognitive decline contributes to the exacerbation of pain perception (Achterberg et al., 2020). In addition, white matter hyperintensities (WMHs) and lacunes in the brain are commonly seen on MRI of patients with CI (Ding et al., 2018; Femir-Gurtuna et al., 2020) and potentially affect the connections in the descending modulatory system of pain, aggravating pain perception (Oosterman et al., 2006).

Moreover, mood disorders, such as depression, anxiety, and sleep disturbance, are usually observed not only in patients with BMS (Adamo et al., 2015, 2018, 2020; Galli et al., 2017) but also in patients with CI (Chan et al., 2020). It is also known that these psychological comorbidities often evolve and are considered as a risk factor for a cognitive decline resulting in dementia (Cerejeira et al., 2012; Whitlock et al., 2017; Chan et al., 2020). Several studies have evaluated the cognitive profile in different NCP conditions such as fibromyalgia (Rodríguez-Andreu et al., 2009), postherpetic neuralgia (Pickering et al., 2014), and chronic back pain (Schiltenwolf et al., 2017) although no studies have been performed on patients with BMS. However, in our clinical practice, the majority of patients with BMS across the years reported the typical subjective experience of CI, particularly concentration difficulties and forgetfulness that still remain despite the ability of the proper treatment to control pain and psychiatric symptoms. Therefore, we supposed to show the possibilities of a cognitive function impairment and to evaluate the potential involvement of domain type and performed a complete cognitive battery test.

This is the first study to evaluate the prevalence of CI in patients with BMS through a comprehensive cognitive assessment taking into account also the psychological profile of patients and by analyzing the potential predictors of a cognitive decline.

Our specific hypothesis states that patients with BMS could show a cognitive performance impairment compared with healthy participants even after its identification for potentially confounding factors. Therefore, the primary outcome of this study is to analyze cognitive and psychological profiles (anxiety, depression, and sleep quality) to report the pain and quality of life in a cohort of patients with BMS compared with a control group of healthy subjects matched for gender, age, and educational level. Our secondary outcome is to identify the predictive risk factors of CI in patients with BMS to evaluate sociodemographic characteristics (age, gender, education, job, and marital status) and health-related factors [body mass index (BMI), disease onset, smoking, alcohol use, sleep duration, physical activity, vascular diseases, and blood biochemical biomarkers], and psychological variables and brain neuroimaging methods measure the age-related white matter changes (ARWMCs) as covariates to account for confounding based on the associations between pain and cognition as reported in previous studies.

## Methods

### Study Design and Participants

An observational case-control study was conducted at the Oral Medicine Department of University of Naples “Federico II” in accordance with the ethical principles of the World Medical Association Declaration of Helsinki and was approved by the Ethical Committee of the University (Approval Number: 251/19—the date of approval was February 20, 2019). The adopted methods conformed with the Strengthening of the Reporting of Observational Studies in Epidemiology (STROBE) guidelines for observational studies (von Elm et al., 2014).

This study was conducted between March 2019 and December 2020. A total of 100 participants aged 55–80 years were recruited, specifically patients suffering from BMS at the first consultation and without previous treatment, and healthy subjects presenting exclusively for dental care during the study period. All consecutive eligible subjects were invited to participate in this study and provided written informed consent. No payment was provided for participation in this study. The recruitment of patients with BMS and healthy subjects was based on a convenience sampling.

The case and the control groups were matched by age, gender, and educational level. In detail, patients with BMS were enrolled first and used to calculate the gender distribution, average age, and educational level; then the control group was recruited to obtain a matched sample.

**Figure 1:**
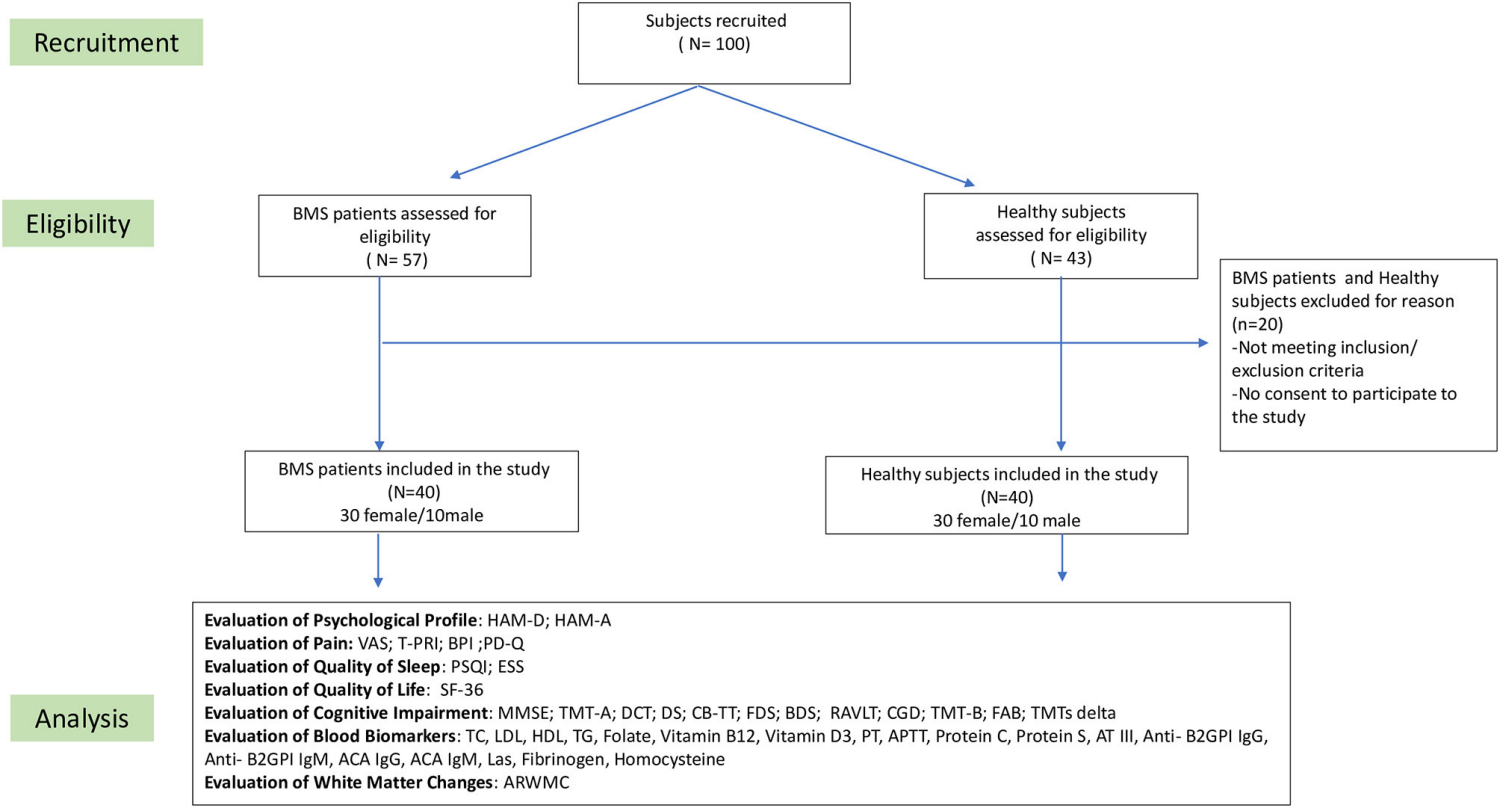
The flowchart of this study.

In the baseline visit (time 0), 57 patients in the study group and 43 in the control group were considered eligible for this study, but only 40 individuals in each group met the inclusion and exclusion criteria. The flowchart of this study is summarized in Figure 1.

The BMS group inclusion criteria were in line with the International Classification of Orofacial Pain (2020) 1st edition:

- patients experiencing the symptoms of oral burning recurring daily for >2 h per day for >3 months without any clinical mucosal alterations,
- patients aged between 55 and 80,
- patients with normal blood test findings (including blood count, blood glucose levels and glycated hemoglobin, serum iron, ferritin, and transferrin),
- patients who are currently not in treatment with psychotropic drugs, and
- patients without any contraindications to MRI scanning (e.g., pacemaker).

The BMS group exclusion criteria were as follows:

- patients suffering from diseases that could be recognized as a causative factor of BMS,
- patients aged <55 and more than 80 years,
- patients unable to understand the questionnaires,
- patients having a history of a psychiatric disorder or a neurological or an organic brain disorder,
- patients undergoing the treatment with psychotropic drugs,
- patients having a history of alcohol or substance abuse,
- patients in treatment with systemic drugs possibly associated with oral symptoms, and
- patients suffering from obstructive sleep apnoa syndrome (OSAS).

The inclusion criteria of a healthy subject were as follows:

- subjects without any lesion of the oral mucosa,
- patients aged <55 and more than 80 years,
- subjects without a psychiatric disorder or a neurological or an organic brain disorder,
- subjects without a history of BMS,
- subjects with normal blood test findings (including blood count, blood glucose levels and glycated hemoglobin, serum iron, ferritin, and transferrin),
- subjects who had not undergone treatment with psychotropic drugs, and
- subjects without any contraindications to MRI scanning (pacemakers or other metal objects).

The exclusion criteria of a healthy subject were as follows:

- subjects with a history of BMS,
- subjects aged <55 and more than 80,
- subjects suffering from a psychiatric disorder, a neurological, or an organic brain disorder, or other conditions possibly resulting in CI,
- subjects unable to understand the questionnaires,
- subjects undergoing treatment with psychotropic drugs,
- subjects having a history of alcohol or substance abuse, and
- patients suffering from OSAS.

### Measures

#### Baseline Clinical Assessment

At admission, the data related to sociodemographic factors were analyzed for each group, including gender, age, years of education, family situation (single, married, divorced, and widowed), and employment (employed, unemployed, and retired). In addition, BMI (calculated as the weight in kilograms divided by the height in square meter), disease onset (in years), sleep duration (in hours), risk factors (current smoking status, alcohol consumption, and physical activity), oral symptoms, systemic diseases, and drug consumption were recorded.

All the patients were evaluated by a multidisciplinary team composed of two oral medicine specialists [Daniela Adamo (DA) and Federica Canfora (FC)] and one board-certified psychiatrist [Giuseppe Pecoraro (GP)] with also a neurology board certification. All of them have at least 5 years of experience in the psychiatric and pain assessment of elderly subjects with chronic orofacial pain. Each subject underwent a careful medical analysis, a general medical examination, an intraoral and extraoral clinical examination, and a psychiatric evaluation. In the first evaluation, the systolic blood pressure (SBP) and diastolic blood pressure (DBP) were measured two times in the right arm using an automatic device after minutes of rest in a seated position. The neuropsychological evaluation was completed using a set of battery scales exploring the neurocognitive, psychological profile and complete pain assessment of patients and healthy subjects; every evaluation of each patient was performed in a designated hospital room by a psychiatrist (GP) in the team, to standardize the clinical procedures. The physician provides the same documentation and a pen to every patient. In their first evaluation, venous blood samples were collected in the morning from all participants who agreed. Cognitive assessment and blood samples were collected on the same day. Within 4 weeks after the baseline examination, all subjects underwent the MRI of the brain using a standard protocol. This choice was made to prevent high levels of anxiety commonly reported by many patients who underwent MRI procedures, which could have an impact on cognitive test performance (van Minde et al., 2014).

Two neuroradiologists [Renato Cuocolo (RC) and Lorenzo Ugga (LU)] rated a white matter change (WMC) severity for the participants of this study using the clinically blinded ARWMC score.

#### Neurocognitive Assessment

**Figure 2:**
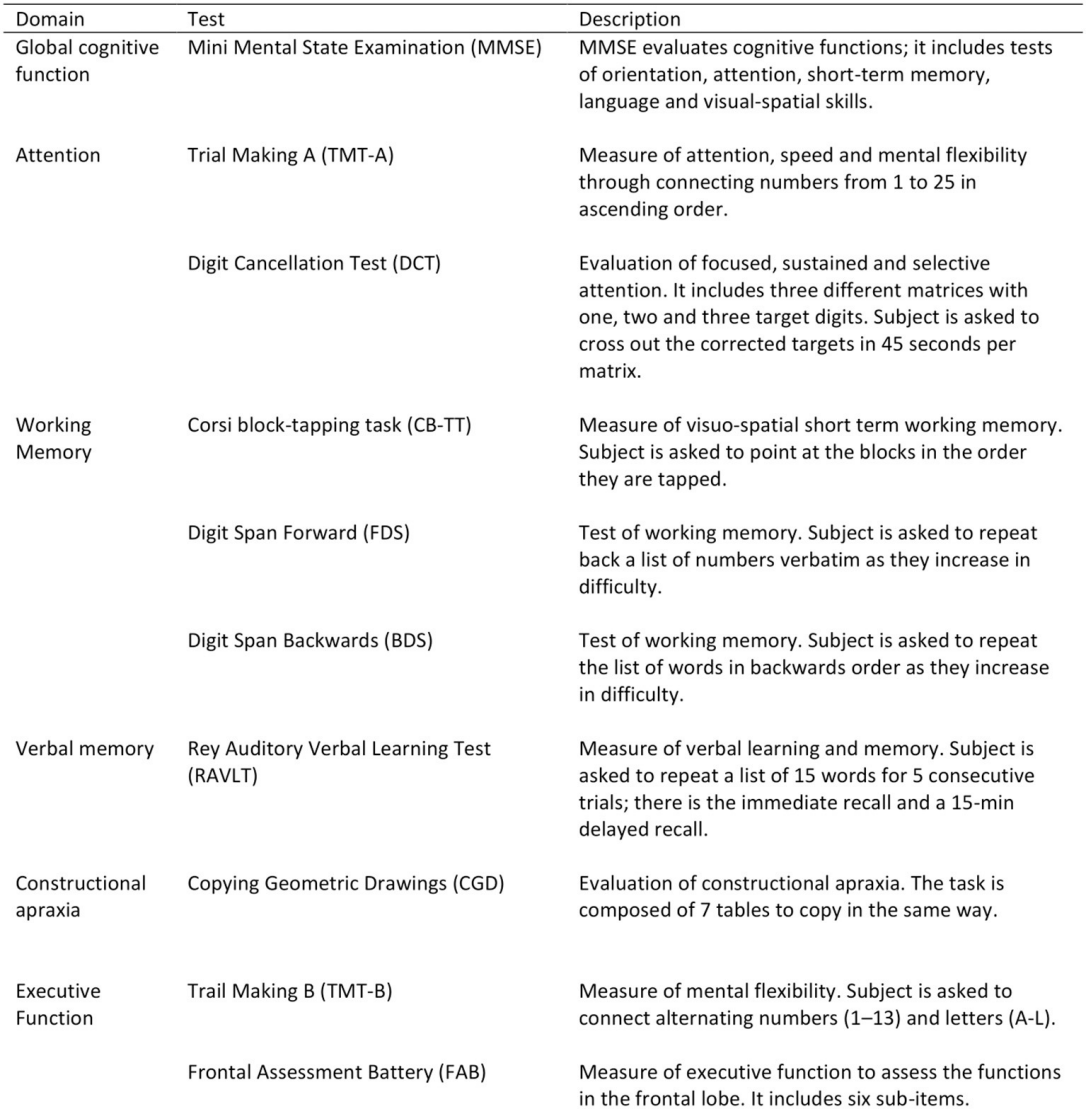
Neurocognitive assessment.

All participants underwent a detailed one-to-one complete cognitive assessment using standardized protocols to assess global mental status, attention, processing speed, working memory, verbal memory, praxis-constructive skills, and executive functions (Figure 2).

The cognitive battery test has included:

- the Mini Mental State Exam (MMSE),
- the Trail Making Test (TMT) part A and part B,
- the Digit Cancellation Test (DCT),
- the Corsi Block-Tapping Task (CB-TT),
- the forward and backward Digit Span Task (FDS/BDS),
- the Rey Auditory Verbal Learning Test (RAVLT),
- the Copying Geometric Drawings Test (CGD),
- the Frontal Assessment Battery (FAB).

All the scales are reviewed for their completeness before collection, administered in their Italian version, and described in detail in the Supplementary Material. The complete cognitive battery examination takes about 80 min to be administered.

#### Psychological Assessment

##### Depression

*The Hamilton rating scale for Depression (HAM-D)* (Hamilton, 1960, 1967) is a clinician-administered depression assessment scale; it contains 21 items pertaining to the affective field. The scores can range from 0 to 54. A score > 7 indicates an impairment. The scores in the range of 7–17 indicate a mild depression, the scores between 18 and 24 indicate a moderate depression, and the scores >24 indicate a severe depression (Morriss et al., 2008).

##### Anxiety

*The Hamilton rating scale for Anxiety (HAM-A)* (Hamilton, 1959) is a clinician-administered anxiety assessment scale. It comprises 14 items to measure both psychic anxiety and somatic anxiety. Each item is scored on a scale of 0–4, a total score <17 indicates a mild severity, 18–24 mild to moderate, and 25–30 moderate to severe (Hamilton, 1967).

##### Sleep

Subjective sleep quality and daytime sleepiness were evaluated using the Pittsburgh Sleep Quality Index (PSQI) and Epworth Sleepiness Scale (ESS), respectively.

The *PSQI* (Buysse et al., 1989) is a self-rated questionnaire, which assesses sleep quality and disturbances over a 1-month time interval generating seven “component” scores (0–3): subjective sleep quality, sleep latency, sleep duration, habitual sleep efficiency, sleep disturbances, the use of sleeping medication, and daytime dysfunction (Carpenter and Andrykowski, 1998). The sum of scores for these seven components yields one global score ranging from 0 to 21. Global scores >5 distinguish the poor sleepers from good sleepers with a high sensitivity (90–99%) and specificity (84–87%) (Curcio et al., 2013).

The *ESS* (Johns, 1991) is a self-administered questionnaire for measuring the general level of daytime sleepiness of a subject. The instrument comprises eight items assessing the propensity for sleep in eight common situations. Subjects rate their likelihood of dosing in each situation on a scale of 0 (would never dose) to 3 (a high chance of dosing). The ESS score (Johns, 1992) is the sum of the eight items, ranging from 0 to 24, with a cut-off value of >10 indicating excessive daytime sleepiness (Vignatelli et al., 2003).

##### Health-related Quality of Life

The *36-Item Short Form Health Survey (SF-36)* (Ware and Gandek, 1998) is a very popular instrument for evaluating health-related quality of life (HRQoL). It is a self-administered questionnaire and comprises 36 questions, which cover the eight domains of health; the physical health measure includes the four scales of physical functioning (PF: 10 items), role physical (RP: 4 items), bodily pain (BP: 2 items), and general health (GH: 5 items); the mental health (MH) measure is composed of vitality (VT: 4 items), social functioning (SF: 2 items), role emotional (RE: 3 items), and MH (5 items). The scores for the SF-36 scales range between 0 and 100, and the total score is a perfect equilibrium between the physical (50%) and mental (50%) components with higher scores indicating a better HRQoL (Jenkinson et al., 1999; Laucis et al., 2015).

#### Pain Assessment

The *Visual Analog Scale (VAS)* (Hayes and Patterson, 1921) is a well-validated unidimensional instrument for the measure of pain intensity (Hawker et al., 2011). The score is determined by measuring the distance on the line between the “no pain” and the mark of a patient mark, providing a range of scores from 0 to 10 (0 = no oral symptoms and 10 = the worst imaginable discomfort).

*The Brief Pain Inventory (BPI)* is a validated and widely used inventory that is developed to assess the severity of pain and the impact of pain on daily functions (Jumbo et al., 2021). It is a 9-item self-administration in which the pain severity is assessed by 4 items, including the worst and least pain in the previous 24 h, pain severity on average, and pain “right now” ranging from 0 (no pain) to 10 (pain as bad as you can imagine).

Pain-related interference rates the degree to which the pain affects the seven domains of functioning (general activity, mood, walking ability, normal work, relations with other persons, sleep, and the enjoyment of life). The score ranges between 0 (does not interfere) to 10 (completely interferes) (Im et al., 2020).

There is no scoring algorithm, but “worst pain” or the arithmetic mean of the four severity items can be used as the measures of pain severity; the arithmetic mean of the seven interference items can be used as a measure of pain interference.

The *Pain DETECT Questionnaire (PD-Q)* is a reliable screening tool with a high sensitivity and specificity for the identification of neuropathic pain (Migliore et al., 2021). It is a self-reported questionnaire and consists of nine items: seven sensory symptom items, including burning, tingling, or prickling sensations, tactile and thermal allodynia, electric shock-like sensations, numbness, and pressure-evoked pain sensation, which are graded from 0 to 5 on a Likert-type scale indicating never to very strongly agree; 1 temporal item on a pain course pattern graded from −1 to +1; and 1 spatial item on pain radiation graded from 0 for no radiation to +2 for radiating pain. The total score calculated from the nine items ranges from −1 to 38, with higher scores indicating the higher levels of neuropathic pain. The developer of the scale has suggested that patients with a score of ≤ 12 have an 85% likelihood of not suffering from neuropathic pain and patients with a score of ≥19 have a 90% likelihood of suffering from neuropathic pain (König et al., 2021).

The *Total Pain Rating Index Questionnaire (T-PRI)* from the short form of the McGill Pain Questionnaire (SF-MPQ) is a measure of the quality of pain and is a multidimensional pain questionnaire, which measures the sensory, affective, and evaluative aspects of the perceived pain (Hawker et al., 2011). It comprises 15 items from the original MPQ, each scored from 0 (none) to 3 (severe). The T-PRI score is obtained by summing the item scores (range 0–45). There are no established critical cutoff points for the interpretation of the scores and, as for the MPQ, a higher score indicates the worse pain.

#### Blood Sampling, Laboratory Tests, and Biochemical Markers

The laboratory test was analyzed from a venous blood sample obtained at morning after a fasting period of about 12 h. The samples were collected in the morning between 8 and 10 a.m., and serum and plasma samples were centrifuged within 30 min and stored at −80°C until being processed. All biochemical analyses were performed with a Roche Modular Analytics System in the Central Biochemistry Laboratory of our Institution.

The analysis included the full blood count, erythrocyte sedimentation rate, glucose and glycosylated hemoglobin, and a thyroid function.

In addition, the evaluation was completed with the following parameters:

- *Blood lipids: total cholesterol* (*TC*; measurement range 160–200 mg/dl), *low-density lipoprotein cholesterol* (*LDL*; measurement range 40–130 mg/dl), *high-density lipoprotein cholesterol* (*HDL*; measurement range 40–80 mg/dl), and *triglycerides* (*TG*; measurement range 60–180 mg/dl) were evaluated using the standard enzymatic-colorimetric method with an autoanalyzer (MIRA-PLUS, Roche, Basel, Switzerland). LDL and HDL cholesterol were determined using a direct method (a homogeneous enzymatic assay for the direct quantitative determination of LDL and HDL cholesterol). HDL was evaluated after the precipitation of apolipoprotein B-containing particles with phosphotungstic acid and a magnesium ion.
- *Folate levels* (measurement range 3.0–16.5 ng/ml), *B12 vitamin* (measurement range 197–866 pg/ml) level, and *D3 vitamin* (measurement range 10–100 ng/ml) *level*.
- *Thrombophilia testing: serum total homocysteine* (*Hcy*; measurement range > 11 μM), *prothrombin time* (*PT*; measurement range:11–13 s), *partial thromboplastin time* (*aPTT*; measurement range 28–40 s), *protein C activity* (measurement range 70–120%) and *protein S activity* (measurement range > 58%), *plasma antithrombin III* (*AT III*; measurement range 70–120%), *anti-*β*2-glycoprotein I antibodies* (*anti*β*2GPI*; IgM and IgG; measurement range <20 U/ml), *anticardiolipin antibodies* (*ACA*; IgM and IgG; measurement range <20 U/ml), *Lupus anticoagulants* (*LAs*; measurement range RATIO <1.20 absent), *fibrinogen* levels (measurement range 160–350 mg/dl). Venous blood was collected in 0.1-M buffered trisodium citrate (bloodcitrate 9:1). Two centrifugation steps (10 min at 3,000 g) were performed to obtain a platelet-poor plasma. The plasma was aliquoted, frozen, and stored at −20°C until use. AT and protein C activities were determined using the Coamatic LR Antithrombin Kit (Chromogenix, Milano, Italy) and the Immunoserum PC Kit (Baxter AG, Vienna, Austria), respectively. The functional activity of protein S was determined using the protein S Reagent Kit (Dade Behring, Marburg, Germany). The presence of LAs was determined using the LA 1 Screening Reagent and LA 2 Confirmation Reagent (Dade Behring, Deerfield, IL, USA). ACA and antiβ2GPI were determined using an ELISA method (Synelisa Cardiolipin antibodies and antiβ2GPI; Pharmacia & Upjohn, Freiburg, Germany; microplate reader, Tecan, Crailsheim, Germany). Fibrinogen was tested on the ACL TOP analyzer (Instrumentation Laboratory, Werfen Group, Bedford, MA, USA). All functional coagulation assays were performed using an autoanalyzer system (BCS, Dade Behring, Marburg, Germany).

### Acquisition of MRI and ARWMC

MRI was performed on a 1.5T Philips Gyroscan Achieva, MRI System (Philips, Best, The Netherlands) using a standard protocol, which included a fluid-attenuated inversion recovery sequence (TR: 8,005 ms, TE: 100 ms, TI: 2,200 ms, matrix: 256 × 192, slice thickness: 5 mm) and a turbo spin echo T2-weighted sequence (TR: 4,400 ms, TE: 100 ms, matrix: 256 × 192, slice thickness: 5 mm). According to the original study of the ARWMC scale, we defined WMC as ill-defined hyperintensities ≥5 mm on both T2-weighted and FLAIR images. This scale grades WMC severity in five brain regions (frontal lobe, parieto-occipital region, temporal lobe, infratentorial region, and basal ganglia) on a four-point scale (score 0 = no lesions, 1 = focal lesions; 2 = beginning confluence of lesions; and 3 = diffuse involvement). The results can be presented as the total score and global score. In this study, the total score was used, representing the sum of scores for each region in both hemispheres, which range from 0 to 30.

### Primary Outcomes

The outcomes evaluated for the primary objective of the study were the presence of CI as measured by MMSE, DCT; FDS and BDS, CBTT, RAVLT immediate and delayed, CGD, FAB, and Trail Making A and B (TMT-A and TMT-B) and the reported pain as measured by VAS, BPI, PD-Q, and T-PRI, the psychological profile as measured by HAM-D, HAM-A, PSQI, ESS, and SF-36 with an aim to detect any potential differences between patients with BMS and healthy subjects.

### Secondary Outcomes (Vascular Disease and Risk Factor Assessment)

The outcomes evaluated for the second objective of this study were the analysis of laboratory tests, including a thrombophilic evaluation, and the examination of ARWMC scores to detect the differences between patients and controls. Finally, we wanted to evaluate the potential predictors for CI in a sample of patients with BMS, so we included the following list of confounding variables associated with CI: age in years, gender (male and female), education (in years), marital status (single, married/partnered, divorced, and widowed), employment status (employed/unemployed), pain (VAS, BPI, PD-Q, and T-PRI), depression (HAM-D), anxiety (HAM-A), sleep disturbance (PSQI and ESS), and HRQoL (SF-36:PF, RP, BP, GH, VT, SF, and RE).

In addition, the following vascular comorbidity variables and other health variables and their correlation with CI were analyzed in the study groups:

- *High blood pressure*: The subject was considered as hypertensive in case of having blood pressure (BP) higher than 140/90 mm Hg (SBP/DBP) according to the current criteria ACCF/AHA for uncomplicated hypertension in the elderly (Messerli et al., 2011) or in case of taking antihypertensives.
- *Obesity*: The subject was considered as obese if they had the BMI values ≥30 kg/m^2^ as indicated by the current AHA/ACC/TOS criteria for obesity in adults (Lavie et al., 2014).
- *Positive history for vascular disease* (i.e., heart attack, atrial fibrillation, and stroke): These diagnoses were made if the subject exhibited a medical documentation certifying a specific diagnosis after admission (Zuin et al., 2021).
- *Dyslipidemia*: For the evaluation of the presence/absence of dyslipidemia; the values of the obtained lipid fractions were dichotomized using the cutoffs for the Metabolic Syndrome recommended by the NCEP-ATPIII (Welty, 2001). In particular, based on these criteria, the following cutoffs were used for the classification of borderline-elevated lipid values: CT ≥ 200 mg/dl; LDL ≥ 130 mg/dl; HDL ≤ 60; TG ≥ 150 mg/dl or if the subjects were in treatment with statins.
- *Hyperhomocysteinemia (HHcy)*: For the evaluation of the presence/absence of HHcy, the cutoff considered in this study was 11 μmol/l in line with the International Consensus statement of AD of 2018, where Hcy >11 μmol/l (Smith et al., 2018; Hsu et al., 2020) was associated with an increase rate of atrophy of the medial temporal lobe and with a greater incidence risk of a cognitive decline (Galton et al., 2001; Smith et al., 2018).
- *Smoking* was assessed *via* the questions on the number of cigarettes smoked in the last 7 days and categorized as never smoker, very light smokers (<5 cigarettes), light smokers (5–10 cigarettes), moderate smokers (10–15 cigarettes), and heavy smokers (>15 cigarettes) (Sabia et al., 2012).
- *Alcohol consumption* was assessed *via* the questions on the number of alcoholic drinks (“measures” of spirits, “glasses” of wine, and “pints” of beer) consumed in the last 7 days and categorized as non-alcoholic (none or <1 unit/week), moderate drinkers (1–14 units/week in women and 1–21 units/week in men), and heavy drinkers (≥15 units in women and ≥21 units in men) (Hagger-Johnson et al., 2013).
- *Physical activity*: The answers of yes were considered if the subject practiced regularly at least 30 min of aerobic activity, three times a week (Lytle et al., 2004).
- *Sleep duration* was assessed *via* a question on the number of hours of sleep during the night in the last 7 days to evaluate whether the patients with BMS suffer due to insufficient (≤ 4 h per night) or excessive (≥10 h per night) sleep duration; as insufficient or excessive sleep duration is actually considered as the risk factors for CI (Ma et al., 2020).

### Statistical Analysis

The statistical analysis was performed using the SPSS software v. 26. Descriptive statistics, including means, SDs, medians, and interquartile range (IQR), were used to analyze the sociodemographic and the clinical characteristics of the two groups. The Pearson Chi-Squared test was used to test the significance differences between the percentages in the two groups.

Differences associated with the values of *p* < 0.05 or 0.01 were considered moderately or strongly significant, respectively.

A *post hoc* power calculation was performed for the Mann–Whitney test. Considering the analysis of different cognitive tests, the effect size ranged from 0.69 to 0.76 for a sample size of 40 participants in each group, with a significance level of 0.05. The power test value (1-Beta) was from 0.91 to 0.97 (the analysis performed *via* the Gpower software).

The scores of ARWMC scale were transformed using a square root arithmetic transformation. The Mann–Whitney *U*-test was employed to test for any differences among blood biochemical markers and ARWMC and to evaluate the recorded medians of the VAS, BPI, PD-Q, T-PRI, HAM-D, HAM-A, PSQI, ESS, SF-36, MMSE, DCT, DS forward, DS backward, CBTT, RAVLT immediate, RAVL delayed, CGD, FAB, TMT-A, TMT-B, and TMT-B-TMT-A in the groups. The values of *p* < 0.05 were considered to reflect a statistical significance.

The Mann–Whitney *U*-test was used to analyze the correlation between a cognitive test and gender, marital status, employment status, smoking and alcohol consumption, physical activity, HHcy, dyslipidemia, essential hypertension, and left temporal and right temporal WMG (LT/RTWMC).

The Spearman test was used to analyze the correlation between a cognitive test and age, years of education, BMI, sleep duration, disease onset, VAS, BPI, PD-Q, T-PRI, HAM-D, HAM-A, PSQI, ESS, and SF-36. The values of *p* < 0.05 were considered to reflect a statistical significance.

Correlation matrices, using the patient group data only, were constructed to identify potential covariates. Finally, multivariate linear regression analyses were computed by entering all the identified variables/predictors of a univariate analysis; unadjusted coefficient estimations were obtained for each significant predictor identified from the correlation analysis. A total of eight models were computed. For each model, we reported the adjusted *R*^2^, which measures the overall goodness of fit adjusted for the number of variables included into the model.

The first model was performed to test the contribution of female gender, HHcy, and hypertension to altered MMSE; the second model was performed to test the contribution of the RP of SF-36 to an impaired FDS; the third model was performed to test the contribution of the smoking status to BDS; the fourth model tested the contribution of employment status and the RP of SF-36 to CBTT; the fifth model tested the contribution of years of education and the T-PRI and the RP of SF-36 to an impaired FAB; the sixth model have evaluated the contribution of years of education and hypertension to an impaired TMT-A; and the seventh model tested the contribution of sleep duration to TMT-B while the eighth model have evaluated the contribution of sleep duration and VAS, respectively, to TMTs-Delta.

## Results

### Sociodemographic Data and Risk Factors

Demographic characteristics, BMI, disease onset, sleep durations, and habits related to the patients with BMS and controls are summarized in Table 1. A total of 80 participants were included in this study, 40 patients with BMS and 40 healthy participants, and no missing data were recorded. Patients with BMS and controls are chosen as a convenient sample for age, gender, and education level, considering the prevalence of BMS in female population and in the elderly. Of these participants, 70% (30) and 30% (10) were women and men for each group, respectively. The mean age of the patients with BMS was 65.63 ± 8.59 with a disease onset of 21.40 ± 25.25.

**Table 1:** Sociodemographic characteristics, body mass index (BMI), disease onset, sleep duration, and habits in patients with burning mouth syndrome (BMS) and control subjects.

**Demographic variables**	**BMS**	**Controls**	***P*-value**
Gender	Frequency (%)	Frequency (%)	
Male	10 (30%)	10 (30%)	1.000
Female	30 (70%)	30 (70%)	
Age (in years)	Mean ± SD	Mean ± SD	
	65.63 ± 8.59	63.73 ± 9.55	0.285
Education (in years)	9.30 ± 5.29	7.104 ± 5.29	0.833
Family situation	Frequency (%)	Frequency (%)	
Single	3 (7.5%)	0 (0%)	0.226
Married	33 (82.5%)	38 (95%)	
Divorced	1 (2.5%)	1 (2.5%)	
Widowed	3 (7.5%)	1 (2.5%)	
Employment	Frequency (%)	Frequency (%)	
Employed	8 (20%)	15 (37.5%)	0.205
Unemployed	20 (50%)	17 (32.5%)	
Retired	12 (30%)	8 (20%)	
Body Mass Index (BMI)	Mean ± SD	Mean ± SD	
	27.51 ± 4.28	26.86 ± 3.50	0.41
Disease onset (months)	21.40 ± 25.25	NA	/
Sleep duration (hours)	6.04 ± 1.30	6.67 ± 1.10	0.020^*^
**Risk factors**	**BMS**	**Controls**	***P*** **-value**
Smoking	Frequency (%)	Frequency (%)	
Never Smokers	30 (75%)	32 (80%)	0.316
Very Light smokers (<5 cigarettes)	4 (10%)	1 (2.5%)	
Light smokers (5–10 cigarettes)	2 (5%)	0 (0%)	
Moderate smokers (10–15 cigarettes)	2 (5%)	3 (7.5%)	
Heavy smokers (>15 cigarettes)	2 (5%)	4 (10%)	
Alcohol use	Frequency (%)	Frequency (%)	
Moderate drinkers (<14 units/week)	8 (20%)	1 (2.5%)	0.013^*^
Not	32 (80%)	39 (97.5%)	
Physical activity			
Yes	14 (35%)	28 (70%)	0.002^**^
No	26 (65%)	12 (30%)	

*A significant difference between the percentages was measured by the Pearson Chi-Squared test*.

* *Significant 0.01 < p ≤ 0.05*,

** *Significant p ≤ 0.01*.

*A significance difference between the means were measured by the t-test*.

^*^Significant 0.01 < p ≤ 0.05,

^**^Significant p ≤ 0.01.

A statistically significant difference was found only in sleep duration, alcohol consumption, and physical activity. Indeed, a patient with BMS was referred to a shorter sleep duration (6.04 ± 1.30; *p* = 0.020^*^), eight patients were moderate drinkers (<14 units/week; *p* = 0.13^*^), and in addition the majority of patients (26; 65%) did not practice regular physical activity (*p* = 0.002^**^).

### Medical Comorbidity and Drug Consumption

The prevalence of systemic diseases and drug consumption is summarized in Table 2. No statistical difference in the prevalence of systemic comorbidities was found between cases and controls. However, statistically significant differences in taking medications were found with a higher consumption of proton pump inhibitors (13, 32.5%; *p* = 0.032^*^), folate (16, 40%; value of *p*- < 0.001^**^), and cholecalciferol (24; 60%; *p* < 0.001^**^) in patients with BMS compared with controls. These results suggested that BMS has already received the supplementation to balance the deficiency of folate and D3 vitamin and to reduce the Hcy serum levels.

**Table 2:** The prevalence of systemic diseases and the drug consumption of patients with BMS and control subjects.

**Systemic diseases**	**BMS Frequency (%)**	**Controls Frequency (%)**	***P*-value**
Essential Hypertension	19 (47.5%)	14 (35%)	0.256
Hypercholesterolemia	14 (35%)	10 (25%)	0.329
Myocardial infarction	0 (0%)	3 (7.5%)	0.077
Atrial fibrillation	2 (5%)	1 (2.5%)	0.556
Hyperhomocysteinemia	22 (55%)	18 (45%)	0.371
Asthma	2 (5%)	3 (7.5%)	0.644
Gastroesophageal reflux disease	8 (20%)	5 (12.5%)	0.363
Endocrine disease	1 (2.5%)	0 (0%)	0.314
Hypothyroidism	7 (17.5%)	6 (15%)	0.762
Benign prostatic hypertrophy	1 (2.5%)	1 (2.5%)	1
**Drug Consumption**	**BMS**	**Controls**	***P*** **-value**
Beta blockers	7 (17.5%)	9 (22.5%)	0.576
Angiotensin receptor blockers	3 (7.5%)	5 (12.5%)	0.456
Diuretics	4 (10%)	7 (17.5%)	0.330
Calcium Channel blockers	4 (10%)	3 (7.5%)	0.692
ACE-inhibitors	7 (17.5%)	4 (10%)	0.330
Simvastatin	12 (30%)	6 (15%)	0.108
Antiplatelets	9 (22.5%)	8 (20%)	0.785
Blood thinner	1 (2.5%)	2 (5%)	0.556
Levothyroxine sodium	1 (2.5%)	5 (12.5%)	0.090
Proton pump inhibitors	13 (32.5%)	5 (12.5%)	0.032^*^
Folic Acid	16 (40%)	2 (5%)	<0.001^**^
Cholecalciferol	24 (60%)	3 (7,5%)	<0.001^**^
Vitamin B12	12 (30%)	6 (15%)	0.108

*A significance difference between the percentages was measured by the Pearson Chi-Squared test*.

* *Significant 0.01 < p ≤ 0.05*,

** *Significant p ≤ 0.01*.

### Oral Symptoms and Sites Involved

The type and location of oral symptoms are shown in Table 3. Statistically significant differences was found between patients and controls. The majority of patients with BMS reported a high prevalence of burning sensation (40; 100%); other frequent symptoms were xerostomia (30; 75%), the change in tongue morphology (62.5%), and dysgeusia (47.5%). The predominant location of pain/burning was tongue (38; 95%) followed by lips (33; 82.5%) and gums (26; 65%).

**Table 3:** The prevalence of oral symptoms and sites involved in patients with BMS and control subjects.

**Oral symptoms**	**BMS frequency (%)**	**Controls frequency (%)**	***P*-value**
Burning	40 (100%)	0	<0.001^**^
Xerostomia	30 (75%)	2 (5%)	<0.001^**^
Dysgeusia	19 (47.5%)	0	<0.001^**^
Sialorrhea	7 (17.5%)	0	0.006^*^
Globus pharyngeus	15 (27.5%)	0	<0.001^**^
Itching	5 (12.5%)	0	0.021^*^
Intraoral Foreign Body Sensation	10 (25%)	0	<0.001^**^
Tingling sensation	11 (27.5%)	0	<0.001^**^
Occlusal Dysesthesia	6 (15%)	0	0.011^*^
Change in tongue morphology	25 (62.5%)	0	<0.001^**^
Oral dyskinesia	3 (7.5%)	0	0.077^*^
Dysosmia	2 (5%)	0	0.152
**Location of Pain/Burning**	**BMS frequency (%)**	**Controls frequency (%)**	***P*** **-value**
Gums	26 (65%)	0	<0.001^**^
Cheeks	26 (65%)	1 (2.5%)	<0.001^**^
Lips	33 (82.5%)	1 (2.5%)	<0.001^**^
Tongue	38 (95%)	0	<0.001^**^
Floor of the Mouth	21 (52.5%)	0	<0.001^**^
Anterior Palate	25 (62.5%)	0	<0.001^**^
Soft Palate	18 (45%)	0	<0.001^**^

*A significant difference between the percentages was measured by the Pearson Chi-Squared test*.

* *Significant 0.01 < p ≤ 0.05*,

** *Significant p ≤ 0.01*.

### Biochemical Blood Markers and ARWMC Scores

Biochemical blood markers are shown in Table 4. There was a statistically significant difference in the plasma level of folic acid (*p* = 0.001^**^), vitamin B12 (*p* = 0.004^*^), and vitamin D3 (*p* = 0.004^*^) between patients and controls. Indeed, patients with BMS showed the higher plasma levels of these vitamins due to the use of therapeutic supplementation.

**Table 4:** Analysis of the biochemical blood markers of patients with BMS and control subjects.

**Biochemical blood markers**	**BMS (Median; IQR)**	**Controls (Median; IQR)**	**P-value**
TC (mg/dL)	209.5 [174.8–233.5]	198 [181.5–214]	0.178
LDL (mg/dL)	126.5 [121–145.3]	125 [112.3–135.5]	0.272
HDL (mg/dL)	55 [45–63.4]	50 [40–56]	0.015
TG (md/dL)	134 [112.5–156]	127.5 [96.3–148.8]	0.128
Folate (ng/mL)	6.5 [5.1–8.7]	4.6 [3.3–5.8]	0.001^**^
Vitamin B12 (pg/dL)	351 [247–446.75]	253 [221–335.3]	0.004^*^
Vitamin D3 (ng/mL)	30.7 [24.5–37.2]	25 [12.3–29.1]	0.004^*^
PT (sec)	11.6 [11–12.6]	11.5 [11–12]	0.478
aPTT (sec)	29 [26.3–31.9]	29.2 [26.8–32.4]	0.560
PROTEIN C (%)	119.5 [103–126.5]	120 [111–137.5]	0.281
PROTEIN S (%)	93.5 [79.3–101.4]	90 [77.8–102.5]	0.693
AT III (%)	99 [95–109.5]	101 [92–112]	0.836
Anti-β2GPI IgG (U/mL)	1.8 [1.4–3.7]	3 [1.5–4]	0.211
Anti-β2GPI IgM (U/mL)	0.9 [0.6–1.2]	1 [0–2.8]	0.291
ACA IgG (U/mL)	2 [1.6–3.1]	3 [2–3.3]	0.299
ACA IgM (U/mL)	2 [1–2.9]	1 [1–3]	0.960
Las (RATIO)	0 [0–0]	0 [0–0]	0.308
Fibrinogen (mg/dL)	323 [297.3–362.7]	363.5 [305–402]	0.055
Homocysteine (uM)	12,6 [9.8–15.5]	10.7 [8.7–14.1]	0.195

*IQR is the interquartile range. A significant difference between medians was measured by the Mann–Whitney test*.

* *Significant 0.01 < p ≤ 0.05*,

** * Significant p ≤ 0.01*.

*TC, Total cholesterol; LDL, Low-density lipoprotein; HDL, High-density lipoprotein; TG, Tryglicerides; PT, Prothrombin time; aPTT, Partial thromboplastin time; AT III, Antithrombin 3; Anti-B2GPI, Anti-B2-glycoprotein 1; ACA, Anticardiolipin antibodies; LAs, Lupus anticoagulants*.

The ARWMC scores are summarized in Table 5. There was a statistically significant difference in the scores of the right (RT; *p* = 0.023^*^) and left temporal lobe (LT; *p* = 0.023^*^) between patients and controls, demonstrating the prevalence of WMC in patients with BMS in the temporal area.

**Table 5:** Analysis of the ARWMC scores of patients with BMS and control subjects.

**ARWMC**	**BMS (Median; IQR)**	**Controls (Median; IQR)**	***P*-value**
LF (Left Frontal)	1 [0–1]	1 [0–1]	0.606
RF (Right Frontal)	1 [0–1]	1 [0–1]	0.504
LPO (Left-Parieto-Occipital)	0 [0–1]	0 [0–1]	0.383
RPO (Right-Parietal-Occipital	0 [0–1]	0 [0–1]	0.557
LT (Left Temporal)	0 [0–0]	0 [0–0]	0.023^*^
RT (Right Temporal)	0 [0–0]	0 [0–0]	0.023^*^
LBG (Left-Basal-Ganglia)	0 [0–0]	0 [0–0]	0.155
RBG (Right-Basal-Ganglia)	0 [0–0]	0 [0–0]	0.155
LINF (Left-Infratentorial)	0 [0–0]	0 [0–0]	0.317
RINF (Right-Infratentorial)	0 [0–0]	0 [0–0]	0.317
Total ARWMC score	2 [0–4.8]	2 [0–4]	0.658

*IQR is the interquartile range. A significant difference between medians was measured by the Mann–Whitney test*.

* *Significant 0.01 < p ≤ 0.05*,

** *Significant p ≤ 0.01*.

*ARWMC, Age related white matter changes; LF, Left frontal; RF, Right frontal; LPO, Left parieto-occipital; RPO, Right parieto-occipital; LT, Left temporal; RT, Right temporal; LBG, Left basal ganglia; RBG, Right basal ganglia; LINF, Left infrantentorial; RINF, Right infratentorial*.

### Psychological Profile

Differences in pain, depression, anxiety, sleep quality, and HRQoL between patients with BMS and controls are shown in Table 6. Regarding pain, BMS suffered the higher levels of pain compared with controls with a statistically significant difference in the median and the IQR of the scores of VAS (*p* < 0.001^**^), BPI severity score (*p* < 0.001^**^), BPI interference score (*p* < 0.001^**^), PD-Q (*p* < 0.001^**^), and T-PRI (*p* < 0.001^**^).

**Table 6:** Analysis of pain, depression, anxiety, sleep quality, and the quality of life of patients with BMS and control subjects.

**Clinical parameters**	**BMS (Median; IQR)**	**Controls (Median; IQR)**	***P*-value**
**VAS**	10 [8–10]	[0–0]	<0.001^**^
**BPI**
Severity score	30 [19.3–39]	2 [0–12.8]	<0.001^**^
Interference score	18 [9–32]	0 [0–8.5]	<0.001^**^
**PD-Q**	8 [4.25–11.75]	0 [0–0]	<0.001^**^
**T-PRI**	5 [3–7.75]	0 [0–0]	<0.001^**^
**HAM-D**	18 [13.25–27.75]	3 [2–5]	<0.001^**^
	**Frequency**	**Frequency**	
Mild (10–17)	9 (22.5%)	5 (12.5%)	<0.001^**^
Moderate (18–24)	11 (27.5%)	0 (0%)	
Severe (>24)	19 (47.5%)	0 (0%)	
**HAM-A**	17 [15–21.5]	3 [3–4.8]	<0.001^**^
	**Frequency**	**Frequency**	
Mild (7–17)	21 (52.5%)	7 (17.5%)	<0.001^**^
Moderate (18–24)	16 (40%)	0 (0%)	
Severe (25–30)	3 (7.5%)	0 (0%)	
**PSQI**	8.50 [4.28–11]	5 [3–7]	<0.001^**^
Subjective sleep quality	1.50 [1–3]	1 [0.25–1]	<0.001^**^
Sleep latency	2 [1–2]	1 [0–1]	<0.001^**^
Sleep duration	1 [0.25–2]	1 [0–1]	0.117
Habitual sleep efficiency	1[0–1]	0 [0–1]	0.231
Sleep disturbances	1 [0–2]	1 [1–1]	0.988
Use of sleeping medication	0 [0–1]	0 [0–0]	<0.001^**^
Daytime dysfunction	1 [0–1]	0.50 [0–1]	0.071
**ESS**	5 [3–7.75]	4 [3–6]	0.149
**SF-36**
Physical functioning (PF)	60 [41.25–100]	95 [75–100]	<0.001^**^
Role physical (RP)	75 [0–100]	100 [56.25–100]	0.027^*^
Bodily pain (BP)	51 [40.25–61]	75 [52–100]	<0.001^**^
General health (GH)	47 [35.50–59]	65 [45–72]	0.007^**^
Vitality (VT)	50 [35–50]	60 [42.50–85]	<0.001^**^
Social functioning (SF)	62 [40.25–75]	87 [75–96.75]	<0.001^**^
Role emotional (RE)	66 [0–100]	100 [66–100]	0.002^**^
Mental health (MH)	48 [40–59]	72 [60–80]	<0.001^**^

*A significant difference between the percentages was measured by the Pearson Chi-Squared test*.

* *Significant 0.01 < p ≤ 0.05*,

** *Significant p ≤ 0.01*.

*IQR is the interquartile range. A significant difference between medians was measured by the Mann–Whitney test*.

^*^Significant 0.01 < p ≤ 0.05,

^**^Significant p ≤ 0.01.

*BMS, Burning mouth syndrome; VAS, Visual analog scale; BPI, Brief pain inventory; PD-Q: PainDETECT Questionnaire; T-PRI, Total Pain Rating Index; HAM-D, Hamilton Rating Scale for Depression; HAM-A, Hamilton Rating Scale for Anxiety; ESS, Epworth Sleepiness Scale; PSQI, Pittsburgh Sleep Quality Index; SF-36, 36 items Short Form Survey*.

Regarding mood disorders, the prevalence of depression and anxiety in patients with BMS was 97.5% (39), whereas in the controls group the prevalence of both disorders were 15% (6) with a statistically significant difference in the medians and the IQR of the scores of HAM-D (*p* < 0.001^**^) and HAM-A (*p* < 0.001^**^) between cases and controls. In addition, the majority of patients with BMS showed a severe depression (19, 47.5%) and mild anxiety (21, 52.5%).

Moreover, 75% (30) of the patients with BMS were poor sleepers (PSQI > 5) with a statistically significant difference in the global PSQI score compared with controls (*p* < 0.001^**^), whereas a statistically significant difference between patients and controls is not found in the ESS score (*p* = 0.149).

A statistically significant difference between patients and controls was found in the medians and IQR of some subitems such as subjective sleep quality, sleep latency, and the use of sleeping medication (*p* < 0.001^**^).

Statistically significant differences were found in the medians and the IQR of all the items of SF-36 (PF: *p* < 0.001^**^; RP: *p* = 0.027^*^; BP: *p* < 0.001^**^; GH: *p*= 0.007^**^; VT: *p* < 0.001^**^; SF: *p* < 0.001^**^; RE: *p* = 0.002^**^; and MH: *p* < 0.001^**^). These results suggested that patients with BMS showed a poor HRQoL compared with controls.

### Cognitive Evaluation

The comparison of the cognitive outcomes of patients with BMS and controls is shown in Table 7.

**Table 7:** The analysis of cognitive tests of patients with BMS and control subjects.

**Cognitive test**	**BMS (median; IQR)**	**Controls (median; IQR)**	***P*-value**
**Global cognitive function**
MMSE	23.35 [21.1–25.2]	25.25 [23.5–26.2]	<0.001^**^
**Attention**
TMT-A	105.50 [79.5–153.8]	74.50 [54–100.5]	<0.001^**^
DCT	46.38 [40.3–49.7]	49.38 [44.4–54.6]	0.011^*^
**Working Memory**
CB-TT	4 [3.8–4.4]	4.63 [4.3–5]	<0.001^**^
FDS	5.83 [4.7–6.1]	6.63 [5.3–7.5]	0.004^*^
BDS	3.48 [2.8–4.3]	3.97 [3.5–4.9]	0.043^*^
**Verbal Memory**
RAVLT immediate	38.05 [30–42.9]	39.05 [30.8–43.3]	0.686
RAVLT delayed	8.25 [5.9–9.8]	8.35 [6.4–9.4]	0.893
**Constructional Apraxia**
CGD	12.95 [11.6–13.5]	19.88 [12.3–13.5]	0.580
**Executive Functions**
TMT-B	214.40 [160.8–263.8]	105 [78.6–144.5]	<0.001^**^
FAB	15.05 [14–16.3]	17.30 [16.4–18.2]	<0.001^**^
TMTs Delta	104 [44.3–157.5]	35 [24.3–59]	<0.001^**^

*IQR is the interquartile range. The significance difference between medians was measured by the Mann–Whitney test*.

* *Significant 0.01 < p ≤ 0.05*,

** *Significant p ≤ 0.01*.

*MMSE, Mini Mental State Examination; DCT, Digit Cancellation Test; DS, Digit Span tests; CBTT, Corsi Block-Tapping Task; RAVLT, Rey Auditory Verbal Learning Test; CGD, Copying Geometric Drawings; FAB, Frontal Assessment Battery; TMTs, Trial Making Tests; FDS, Forward Digit Span; BDS, Backwards Digit Span*.

All patients with BMS showed an impairment in at least one cognitive domain.

The median and IQR in the majority of cognitive tasks show a statistically significant difference between patients with BMS and controls such as in MMSE (*p* < 0.001^**^), TMT-A (*p* < 0.001^**^), DCT (*p* = 0.011^*^), CB-TT (*p* < 0.001^**^), TMT-B (*p* < 0.001^**^), TMTs-Delta (*p* < 0.001^**^), FAB (*p* < 0.001^**^), FDS (*p* = 0.004^*^), and BDS (*p* = 0.043^*^). No statistically significant differences were found in the two groups in the task evaluating verbal memory (RAVLT immediate; *p* = 0.686 and RAVLT delayed; *p* = 0.893) and praxis-constructive skills (CGD; *p* = 0.580).

Therefore, patients with BMS showed a decrease in the global cognitive function with the lower scores of MMSE [23.35 (21.1–25.2)]; a decrease of attention with the higher scores of TMT-A [105.50(79.5–153.8)] and the lower scores of DCT [46.38(40.3–49.7)]; a decrease of working memory with the lower scores of CB-TT [4(3.8–4.4)], FDS [5.83(4.7–6.1)] and BDS [3.48(2.8–4.3)]; and a decrease of executive functions with a higher score of TMT-B [214.40(160.8–263.8)] and TMTs-Delta [104(44.3–157.5)] and the lower scores of FAB [15.05(14–16.03)] compared with controls.

Tables 8, 9 show a correlation analysis between cognitive tests and quantitative and qualitative predictors, respectively, in the case group.

**Table 8:** Correlation analysis between cognitive tests and quantitative predictors in patients with BMS.

**Predictors/Cognitive test**	**MMSE**	**DCT**	**FDS**	**BDS**	**CBTT**	**FAB**	**TMT-A**	**TMT-B**	**TMTs delta**
	***ρ (p-value)***	***ρ (p-value)***	***ρ (p-value)***	***ρ (p-value)***	***ρ (p-value)***	***ρ (p-value)***	***ρ (p-value)***	***ρ (p-value)***	***ρ (p-value)***
Age	−0.144 (0.377)	−0.128 (0.430)	0.134 (0.410)	−0.038 (0.815)	−0.023 (0.889)	−0.093 (0.567)	0.276 (0.085)	0.147 (0.367)	0.023 (0.886)
Years of education	0.224 (0.164)	0.010 (0.951)	0.212 (0.188)	0.281 (0.079)	−0.074 (0.649)	0.382 (0.015^*^)	−0.445 (0.004^**^)	−0.277 (0.084)	−0.025 (0.879)
Body Mass Index (BMI)	−0.208 (0.198)	−0.142 (0.383)	0.046 (0.780)	0.049 (0.764)	−0.051 (0.754)	−0.035 (0.832)	−0.041 (0.803)	−0.09 (0.582)	−0.053 (0.745)
Sleep duration (hours)	−0.028 (0.865)	0.297 (0.063)	0.153 (0.347)	0.008 (0.961)	0.01 (0.951)	−0.042 (0.797)	−0.198 (0.220)	−0.355 (0.024^*^)	−0.319 (0.045^*^)
Disease onset (months)	−0.207 (0.201)	−0.122 (0.454)	0.232 (0.151)	0.087 (0.595)	−0.195 (0.228)	−0.013 (0.935)	−0.085 (0.601)	−0.091 (0.575)	−0.022 (0.894)
VAS	−0.277 (0.084)	−0.053 (0.747)	−0.099 (0.543)	−0.264 (0.100)	−0.289 (0.070)	−0.261 (0.103)	0.013 (0.937)	0.23 (0.154)	0.367 (0.020^*^)
T-PRI	0.188 (0.246)	−0.097 (0.553)	0.185 (0.254)	−0.003 (0.983)	0.096 (0.555)	0.326 (0.040^*^)	0.106 (0.516)	0.093 (0.569)	−0.011 (0.947)
PD-Q	0.074 (0.650)	0.027 (0.867)	0.3 (0.060)	0.194 (0.231)	−0.051 (0.756)	0.163 (0.315)	−0.008 (0.959)	0.096 (0.556)	0.053 (0.747)
BPI severity	−0.078 (0.633)	−0.037 (0.821)	0.032 (0.846)	0.172 (0.288)	−0.212 (0.188)	0.029 (0.860)	−0.108 (0.507)	−0.167 (0.302)	0.001 (0.993)
BPI interference	−0.106 (0.514)	−0.230 (0.153)	−0.049 (0.764)	−0.004 (0.980)	−0.163 (0.316)	−0.073 (0.653)	0.077 (0.636)	0.137 (0.399)	0.260 (0.106)
HAM-D	−0.041 (0.803)	−0.023 (0.888)	0.066 (0.688)	0.079 (0.628)	−0.216 (0.180)	−0.106 (0.517)	0.07 (0.669)	0.044 (0.790)	0.058 (0.722)
HAM-A	−0.043 (0.795)	−0.051 (0.754)	0.115 (0.479)	0.23 (0.153)	−0.014 (0.933)	−0.03 (0.855)	0.199 (0.219)	0.129 (0.428)	0.11 (0.50)
PSQI	0.138 (0.394)	−0.303 (0.057)	−0.013 (0.936)	0.066 (0.685)	0.001 (0.998)	0.139 (0.391)	0.183 (0.258)	0.188 (0.245)	0.167 (0.304)
ESS	−0.001 (0.994)	0.181 (0.264)	0.187 (0.247)	−0.082 (0.615)	−0.054 (0.740)	0.155 (0.338)	−0.232 (0.150)	−0.215 (0.183)	−0.036 (0.827)
SF-36 PF	0.022 (0.891)	0.179 (0.270)	−0.213 (0.186)	−0.119 (0.464)	0.176 (0.278)	0.042 (0.799)	−0.058 (0.720)	−0.093 (0.568)	−0.001 (0.996)
SF-36 RP	−0.192 (0.235)	−0.046 (0.776)	−0.381 (0.015^*^)	−0.190 (0.241)	0.209 (0.195)	−0.315 (0.048^*^)	−0.015 (0.925)	0.100 (0.541)	0.140 (0.387)
SF-36 BP	0.076 (0.643)	0.164 (0.313)	−0.140 (0.387)	−0.093 (0.570)	0.133 (0.412)	0.008 (0.963)	−0.250 (0.120)	−0.237 (0.142)	−0.064 (0.694)
SF-36 GH	−0.139 (0.392)	0.117 (0.472)	−0.305 (0.055)	−0.043 (0.793)	0.292 (0.067)	−0.251 (0.118)	−0.126 (0.438)	−0.073 (0.654)	0.029 (0.858)
SF-36 VT	−0.203 (0.210)	0.048 (0.767)	−0.005 (0.976)	−0.205 (0.206)	0.073 (0.655)	−0.275 (0.086)	−0.030 (0.854)	0.014 (0.934)	0.080 (0.625)
SF-36 SF	0.057 (0.726)	0.077 (0.636)	−0.134 (0.410)	0.185 (0.253)	0.141 (0.386)	−0.010 (0.953)	−0.243 (0.131)	−0.231 (0.151)	−0.040 (0.808)
SF-36 RE	0.040 (0.807)	0.051 (0.754)	−0.196 (0.224)	0.062 (0.703)	0.321 (0.043^*^)	−0.177 (0.274)	−0.118 (0.468)	−0.044 (0.786)	0.052 (0.749)
SF-36 MH	−0.080 (0.622)	0.113 (0.488)	0.041 (0.800)	−0.177 (0.274)	0.113 (0.486)	−0.249 (0.121)	−0.012 (0.939)	−0.025 (0.880)	0.017 (0.916)

*ρ is Spearman's correlation coefficient. p-value—*

* *Significant 0.01 < p-value ≤ 0.05*.

** *Significant p-value ≤ 0.01*.

*BMS, Burning mouth syndrome; VAS, Visual analog scale; BPI, Brief pain inventory; PD-Q, PainDETECT Questionnaire; T-PRI, Total Pain Rating Index; HAM-D, Hamilton Rating Scale for Depression; HAM-A, Hamilton Rating Scale for Anxiety; ESS, Epworth Sleepiness Scale; PSQI, Pittsburgh Sleep Quality Index; SF-36, 36 items Short Form Survey. PF, Physical function; RP, Role physical, BP, Bodily pain, GH, General health; VT, Vitality; SF, Social functioning; RE, Role-emotional; MH, Mental health*.

**Table 9:** Correlation analysis between cognitive tests and qualitative predictors in patients with BMS.

**Predictors/Cognitive tests**	**MMSE**		**DCT**		**FDS**		**BDS**		**CBTT**		**FAB**		**TMT-A**		**TMT-B**		**TMTs delta**	
	***Median [Q1:Q3]***	***p-value***	***Median [Q1:Q3]***	***p-value***	***Median [Q1:Q3]***	***p-value***	***Median [Q1:Q3]***	***p-value***	***Median [Q1:Q3]***	***p-value***	***Median [Q1:Q3]***	***p-value***	***Median [Q1:Q3]***	***p-value***	***Median [Q1:Q3]***	***p-value***	***Median [Q1:Q3]***	***p-value***
Gender																		
*Female* *Male*	23 [21;25] 25.2 [24.9;26]	0.040^*^	47.1 [40.7;49.6] 44.6 [43.4;46.6]	0.839	5.85 [4.94;6.20] 5 [4.38;5.31]	0.206	3.52 [2.83;4.42] 3.05 [2.98;3.34]	0.557	4 [3.75;4.31] 4 [3.88;4.25]	0.855	15 [13.8;16.2] 15 [14.9;15.9]	0.543	106 [80.5;154] 116 [96.2;136]	0.822	213 [162;263] 234 [184;261]	0.822	106[44.8;158] 92.5[64.5;112]	0.417
Marital status																		
*Married* *Not married*	23.9 [21;25.2] 23.3 [22.7;23.7]	0.748	45.8[42.2;49.8] 48[39.8;48.8]	0.776	5.87 [5.12;6.13] 4.65 [4.43;5.78]	0.170	3.52 [2.97;4.19] 3.1 [2.84;4.01]	0.859	4 [3.75;4.25] 4.25 [3.5;4.62]	0.801	15.1 [14.2;16.2] 14.7 [13.2;15.8]	0.656	104 [81;130] 107 [90;183]	0.476	212 [160;253] 253 [218;303]	0.270	102[45;156] 108[75;154]	0.631
Employment status																		
*Employed* *Not employed*	23 [21.4;24.7] 23.6 [21;25.2]	0.766	48.1 [45.3;49.5] 45 [39.6;49.6]	0.190	4.88 [4.42;6] 5.85 [5.06;6.48]	0.104	3.52 [3.08;4.11] 3.31 [2.84;4.4]	0.913	4.25 [4.25;4.5] 3.75 [3.75;4.19]	0.013^*^	14.6 [14;15.8] 15.2 [14.2;16.3]	0.453	140 [108;169] 93 [76.8;124]	0.051	213 [184;244] 216 [161;271]	0.864	45.5[26.2;106] 106[74.5;158]	0.126
Smoking status																		
*Smoker* *Not smoker*	23.2 [21.4;24.9] 23.6 [21;25.2]	0.975	47.6 [41;51.5] 45.5 [41.3;49.5]	0.938	5.85 [5.16;6.11] 5.81 [4.73;6.32]	0.888	3.76 [3.46;4.64] 3.08 [2.77;4.10]	0.038^*^	4.25 [3.81;4.25] 4 [3.75;4.44]	0.670	14.8 [14;15.9] 15 [14.3;16.3]	0.606	115 [92;124] 98 [79.5;154]	0.864	239 [158;279] 213 [168;253]	0.779	130[42;168] 104[45.5;146]	0.542
Alcohol use																		
*Yes* *No*	23.6 [21.7;24.2] 23.2 [21.2;25.2]	0.799	48.4 [47.2;49.8] 44.8 [39.9;49.6]	0.272	5.22 [4.82;5.94] 5.85 [4.94;6.20]	0.352	3.06 [2.26;3.70] 3.52 [2.94;4.51]	0.205	4 [3.75;4.62] 4 [3.75;4.25]	0.620	14.8 [14.1;15.6] 15.2 [14.1;15.6]	0.685	112 [92;140] 104 [78.2;153]	0.398	234 [185;263] 213 [156;261]	0.510	97[60.8;156] 104[44.8;153]	0.774
Physical activity																		
*Yes* *No*	23.3 [21.4;25] 23.6 [20.8;25.2]	0.955	48.6 [44.2;51.4] 44.8 [39.6;49.3]	0.144	5.56 [4.64;5.87] 5.96 [5.04;6.62]	0.097	3.31 [2.98;3.98] 3.52 [2.84;4.57]	0.590	4.25 [4;4.69] 3.75 [3.75;4.25]	0.057	15.1 [14.3;15.8] 15 [13.6;16.3]	0.766	85.5 [66.5;124] 115 [90;156]	0.100	194 [166;216] 240 [168;281]	0.212	100[52.8;143] 104[44.8;158]	0.887
Hyperhomocysteinemia																		
*Yes* *No*	24.5 [22.7;25.2] 21.2 [20;24.8]	0.027^*^	46.4 [41.8;49.4] 46.4 [40.4;51.4]	0.849	5.85 [4.75;6.32] 5.50 [4.98;6]	0.446	3.48 [2.84;4.44] 3.36 [2.98;3.98]	0.796	4 [3.75;4.5] 4 [3.5;4.25]	0.371	15.2[14.6;16.7] 14.6[13.4;15.6]	0.070	105 [81.2;154] 106 [81.8;129]	0.968	216 [152;260] 214 [183;260]	0.828	100[37.2;158] 107[54.2;153]	0.488
Hypercholesterolemia																		
*Yes* *No*	24.8 [20.8;25.2] 23 [21.2;25.2]	0.887	45.9 [40.2;48.9] 46.5 [43.4;50.6]	0.660	5.83 [5.20;6.32] 5.83 [4.73;6.13]	0.777	3.14 [2.87;4.32] 3.52 [2.87;4.17]	0.744	4 [3.75;4.19] 4.12 [3.75;4.44]	0.742	15[14.3;16.3] 15.1[14;16.1]	0.620	104 [81.8;145] 107 [81;147]	0.691	228 [168;281] 214 [158;252]	0.419	142[74.5;159] 102[44.2;141]	0.269
Essential Hypertension																		
*Yes* *No*	22.7 [20.4;24.8] 24.3 [22.4;25.2]	0.045^*^	47 [42.6;49.1] 44.8 [40;51]	0.882	5.87 [4.59;6.45] 5.83 [5.04;6.04]	0.807	3.08 [2.86;3.70] 3.87 [2.84;4.5]	0.244	4.25 [3.75;4.5] 4 [3.75;4.25]	0.345	14.9[13.9;16.2] 15.2[14.2;16.1]	0.607	153 [91;168] 90 [76;114]	0.010^*^	216 [173;346] 204 [149;251]	0.155	86[45.5;172] 108[45;151]	0.935
(RTWMC/LTWMC)																		
*Yes* *No*	24.7 [21.8;25.2] 23.2 [21.2;25]	0.628	44.8 [39.8;48.4] 48 [42.2;49.5]	0.555	5.87 [4.52;6.39] 5.83 [5.04;6.13]	0.820	3.08 [2.90;4.42] 3.52 [2.79;4.19]	0.739	4 [3.75;4.25] 4 [3.75;4.5]	0.902	15 [13.7;16.7] 15.1 [14.2;16.1]	0.976	104 [83.5;179] 107 [81;130]	0.661	251 [170;258] 212 [163;264]	0.774	99[39.5;129] 106[54;158]	0.422

*Median [First Quartile Q1; Third Quartile Q3]. Wilcoxon–Mann–Whitney Test. p-value—*

* *Significant 0.01 < p-value ≤ 0.05*.

** *Significant p-value ≤ 0.01*.

*RTWMC, Right temporal white matter changes; LTWMC, Left temporal white matter changes*.

Specifically, a statistically significant positive correlation was found between years of education and FAB (*p* = 0.015^*^), whereas a statistically negative correlation was found between years of education and TMT-A (*p* = 0.004^**^) taking into account that the higher level of education corresponds to the higher scores in FAB and the lower level of education corresponding to the worst performance in TMT-A. In addition, FAB was correlated with T-PRI (*p* = 0.048^*^) and RP (*p* = 0.043^*^). Moreover, RE was positively correlated with CBTT (*p* = 0.043^*^) showing that better RE score corresponds to the higher scores in visual working memory.

Sleep duration was negatively correlated with TMT-B (*p* = 0.024^*^) and TMTs-Delta (*p* = 0.045^*^), therefore short sleep was associated with a decrease of executive functions with the higher scores in TMT-B and TMTs-Delta. In addition, TMTs-Delta was correlated with VAS (*p* = 0.020^*^) suggesting that a higher level of the intensity of pain reflects the higher scores of TMTs-Delta.

Mini Mental State Examination was correlated with gender (*p* = 0.040^*^); HHcy (*p* = 0.027^*^) and hypertension (*p* = 0.045^*^) suggesting that female patients with hypertension showed a lower score in MMSE with an impairment in the global cognitive function; instead, strangely, HHcy reflects the higher scores of MMSE. BDS was correlated with smoking, therefore smokers achieved higher BDS (3.76 vs. 3.08; *p* = 0.038^*^) with a better working memory. CBTT was correlated with employment status, with employed patients showed a better working memory with a higher score in CBTT (4.25 vs. 3.75; *p* = 0.013^*^). In addition, TMT-A was correlated with hypertension (*p* = 0.010^*^), therefore BMS hypertensive patients have a higher score in TMT-A and suffer from a decrease of attention.

A multivariate linear regression analysis between cognitive tests and predictors is shown in Table 10. The first model (MMSE model) testing the contribution of female gender, HHcy, and hypertension to MMSE showed that the MMSE was negatively correlated with female gender and hypertension (*p* = 0.037^*^; *p* = 0.039^*^) and positively correlated with HHcy (*p* = 0.019^*^), resulting in a strongly significant increase in the coefficient of determination (*R*^2^) (DR2 = 26.9%, *p* = 0.002^**^). The second model (FDS model) tests the contribution of the RP of SF-36 to FDS; RP was negatively correlated to FDS although the result was not statistically significant (*p* = 0.060); indeed there was not a significant increase in the *R*^2^ (DR2 = 8.9%, *p* = 0.060). The third model (BDS model) tests the contribution of the smoking to FDS; smoking was positively correlated to BDS but the result was not statistically significant (*p* = 0.078); indeed no significant increase in the *R*^2^ (DR2 = 8.9%, *p* = 0.060) was found. The fourth model (CBTT model) testing the contribution of employment status and the RE of SF-36 to CBTT showed that CBTT was positively correlated with employed patients (*p* = 0.038^*^) and with RP (*p* = 0.27), resulting in a significant increase in the coefficient of determination *R*^2^ (DR2=12.4%, *p* = 0.033^*^).

**Table 10:** Multilinear regression analysis predicting impaired cognitive test in patients with BMS.

**Cognitive test**	**Predictors**	**β**	**SE**	***p*-value**	***R*^**2**^ (*p*-value)**
MMSE	Gender: *Female*	−2.66	1.23	0.037^*^	26.9 (0.002^**^)
	Hyperhomocysteinemia	1.83	0.74	0.019^*^	
	Hypertension	−1.57	0.73	0.039^*^	
FDS	Sf-36: *RP*	−0.01	0	0.060	8.9 (0.060)
BDS	Smoking status: *Smoker*	0.75	0.41	0.078	7.9 (0.078)
CBTT	Employment status: *Employed*	0.45	0.21	0.038^*^	12.4 (0.033^*^)
	Sf-36: *RE*	0	0	0.27	
FAB	Years of education	0.11	0.05	0.042^*^	22.9 (0.006^**^)
	T-PRI	0.07	0.04	0.072	
	SF-36: *RP*	−0.01	0	0.363	
TMT-A	Years of education	−3.05	1.43	0.039^*^	24.5 (0,002^**^)
	Hypertension	31.83	14.95	0.04^*^	
TMT-B	Sleep duration (hours)	−24.6	10.55	0.025^*^	12.5 (0.025^*^)
TMTs Delta	Sleep duration (hours)	−14.9	7.94	0.068	13.4 (0.026^*^)
	VAS	9.86	5.59	0.086	

*SE of beta estimates. p-values were obtained by hypothesis test on regression coefficients*.

* *Significant 0.01 < p-value ≤ 0.05*.

** *Significant p-value ≤ 0.01*.

*BMS, Burning mouth syndrome; VAS, Visual analog scale; T-PRI, Total Pain Rating Index; SF-36: 36 items Short Form Survey; RF, role functioning; RP, role physical*.

The fifth model (FAB model) testing the contribution of years of education and the T-PRI and the RP of SF-36 to FAB showed that FAB was positively correlated with years of education (*p* = 0.042^*^), T-PRI (*p* = 0.072^*^) and negatively correlated with RP (*p* = 0.363), resulting in a strongly significant increase in the *R*^2^ (DR2 = 22.9%, *p* = 0.006^**^). The sixth model (TMT-A model) testing the contribution of years of education and hypertension to TMT-A showed that TMT-A has, respectively, a negative and positive correlation with years of education (*p* = 0.039^*^) and hypertension (*p* = 0.04^*^), resulting in a strongly significant increase in the *R*^2^ (DR2 = 24.5%, *p* = 0.002^**^). The seventh model (TMT-B model) testing the contribution of sleep duration to TMT-B showed that TMT-B was negatively correlated with sleep duration (*p* = 0.025^*^), resulting in a significant increase in the *R*^2^ (DR2=12.5%, *p* = 0.025^*^). The eighth model (TMTs-Delta model) testing the contribution of sleep duration and VAS to TMTs-Delta showed that TMTs-Delta was negatively and positively correlated with sleep duration (*p* = 0.068) and VAS (*p* = 0.086), respectively, resulting in a significant increase in the *R*^2^ (DR2=13.4%, *p* = 0.026^*^). Therefore, hypertension in female patients with BMS showed a worst score in MMSE and could be at risk to develop CI as well as patients who sleep less; indeed, the reduction of sleep duration was associated with the higher scores of TMT-B and TMTs-Delta, resulting in the impairment of executive functions. Moreover, BMS with a higher level of education showed better scores in some cognitive tasks such as FAB and TMT-A, resulting in a better level of attention and cognitive functions; moreover, employed patients with BMS showed the higher scores in CB-TT, resulting in better working memory performances.

## Discussion

Patients affected by NCP are considered to be at high risk to develop a cognitive decline, especially in the domains of attention, working memory, and executive functions (Moriarty et al., 2011; Moriarty and Finn, 2014), which leads to the poor quality of life, difficulty in adherence to medications, increasing the risk of mortality, and health resource utilization (O'Connor, 2009; Colloca et al., 2017).

Burning mouth syndrome is a complex chronic orofacial pain disorder frequently associated with several psychiatric comorbidities such as anxiety, depression, and sleep disturbances (Kim et al., 2020; Pereira et al., 2020) while CI have never been evaluated in a complete way. Only one study is found in the literature by Kim et al. (2020), in an observational but a retrospective study, suggested that patients with BMS were not associated with an increase in the incidence of dementia and Parkinson's disease based on the diagnosis performed in ~10 years by physicians, moreover, they could not assess whether the development of both conditions may be the consequence or were involved in the onset of BMS (Kim et al., 2020). Instead, case-control or prospective studies have never been carried out with an aim to establish if patients with BMS may suffer of CI as well as mood disorders. The results of this study supported our suggestions because patients with BMS showed a mild cognitive impairment (MCI) with a decrease in global cognitive functions, elective attention, sustained attention, cognitive flexibility, working memory, and executive functions while verbal memory and praxis constructive skills were preserved in comparison with controls. The term MCI is suitable for patients with BMS with a cognitive decline as it is generally used for all non-demented individuals with memory impairment more often than expected for their age and that could be at risk to develop AD (Christa Maree Stephan et al., 2013; Breton et al., 2019).

In this study, patients with BMS showed the higher intensity and the quality of pain with a higher interference score in daily activities with a poor HRQoL compared with healthy subjects; the prevalence of depression, anxiety, and SD were very high, 97% (39) and 75% (30), respectively.

In line with previous studies on chronic pain and CI (Cao et al., 2019; Zelaya et al., 2020), we have analyzed all the predictors that generally affects cognitive performance.

A correlation between education and FAB and TMT-A was found; therefore, patients with a higher level of education have showed better attention, speed, mental flexibility, executive functions compared with patients with a lower level of education. These results were confirmed using a multiple linear regression where the years of education represent the most important predictor (FAB: *p* = 0.042^*^; TMT-A: *p* = 0.039^*^). These results are in line with the current literature in which the early life factors, such as less education, affect the resulting cognitive reserve (Livingston et al., 2020).

In addition, patients with better emotional functions showed a better visuospatial working memory. Moreover, a shorter sleep duration affects the mental flexibility and the executive functions of patients with BMS.

Short and long sleep duration, poor sleep quality, and insomnia were associated with a higher risk of CI (Shi et al., 2018) but wherefore sleep potentially affect CI remains unclear. Additionally, it seems that sleep–wake cycle dysregulation can be the cause of the pathophysiological processes of the brain, reducing the activation of glymphatic clearance pathways, increasing brain inflammation, and promoting β-amyloid deposition (Spira et al., 2013; Macêdo et al., 2018). Moreover, a new onset of late-life sleep disturbance, a few years before clinical dementia, might be part of the natural history of the dementia syndrome (Ma et al., 2020).

From the analysis of multiple linear regression, female gender, HHcy, and hypertension could explain 26.9% of the MMSE variance suggesting that female patients with hypertension could be at risk to develop an impairment in the global cognitive function (Smith et al., 2018; Avan and Hachinski, 2021).

Strangely, HHcy, which is considered as a risk for CI in previous studies about another type of NCP (Ansari et al., 2014; Hsu et al., 2020), seems to be a protective factor as well as the occupation because employed patients showed a better working memory (Silvaggi et al., 2020). These results confirm those of previous studies where mental activity, in general, might improve a cognitive function (Jak, 2012; Krell-Roesch et al., 2019). Indeed, a 12-year study on 1,658 people found that old retirement age was associated with the lower risk of dementia (Grotz et al., 2016).

Analyzing all predictors and considering comprehensively the neuropsychiatric assessment, it is possible to consider that female patients with hypertension, a lower level of education, unemployed, and a short sleep duration may be at higher risk to develop a cognitive decline compared with patients without these predictors.

These results are in line with the current literature in which the cognitive reserve in women is limited due to less education and unemployment, therefore older women are more likely to develop CI than men at the same age (Laws et al., 2016; Sohn et al., 2018).

In this study, hypertension was a predictor of CI according to the study of McGrath et al (2017), and persisted elevated blood pression was associated with an increased risk of developing dementia over a 18-year follow-up period (McGrath et al., 2017). Moreover, hypertension was associated with reduced brain volumes and increased WMCs and potentially can cause VD (Beauchet et al., 2013; Avan and Hachinski, 2021).

Strangely, despite the results of finding a statistically significant difference between patients and controls and the presence of literature studies, we did not find any correlation between CI and mood disorders, pain, obesity, alcohol use, physical activity, HHcy, and WMCs.

Taking into account these results, it is not possible to exclude that the union and the endurance of multiple predictors might affect the brain health in patients with BMS. In particular, both hypertension and HHcy could induce cerebral hypoperfusion to cause WMCs, which are predictive for CI and, further, this might have an impact on worsening pain perception.

Indeed, WMCs represent an incomplete ischemia mainly related to cerebral small vessel arteriolosclerosis contributing to VD but also associated with the pathogenesis of AD because ischemic insults or cerebrovascular insufficiency leads to an increased expression of amyloid precursor protein (Lee et al., 2005). It has to be considered that WMCs may progress or even regress over time by monitoring modifiable metabolic and vascular factors such as hypertension, cholesterol, smoking, and Hcy level (Alfaro et al., 2018). Previous studies suggested that a patient with mild CI with the lower scores on a cognitive test and higher ARWMC scores develop AD in around 18 months (Salthouse, 2012; Femir-Gurtuna et al., 2020). Therefore, the evaluation across the time of these scores together with vascular risk factors in promoting dementia could be important even more in patients with BMS because pain could be a further risk for dementia as suggested in previous studies (Ferri et al., 2020; McFarlane et al., 2020).

**Figure 3:**
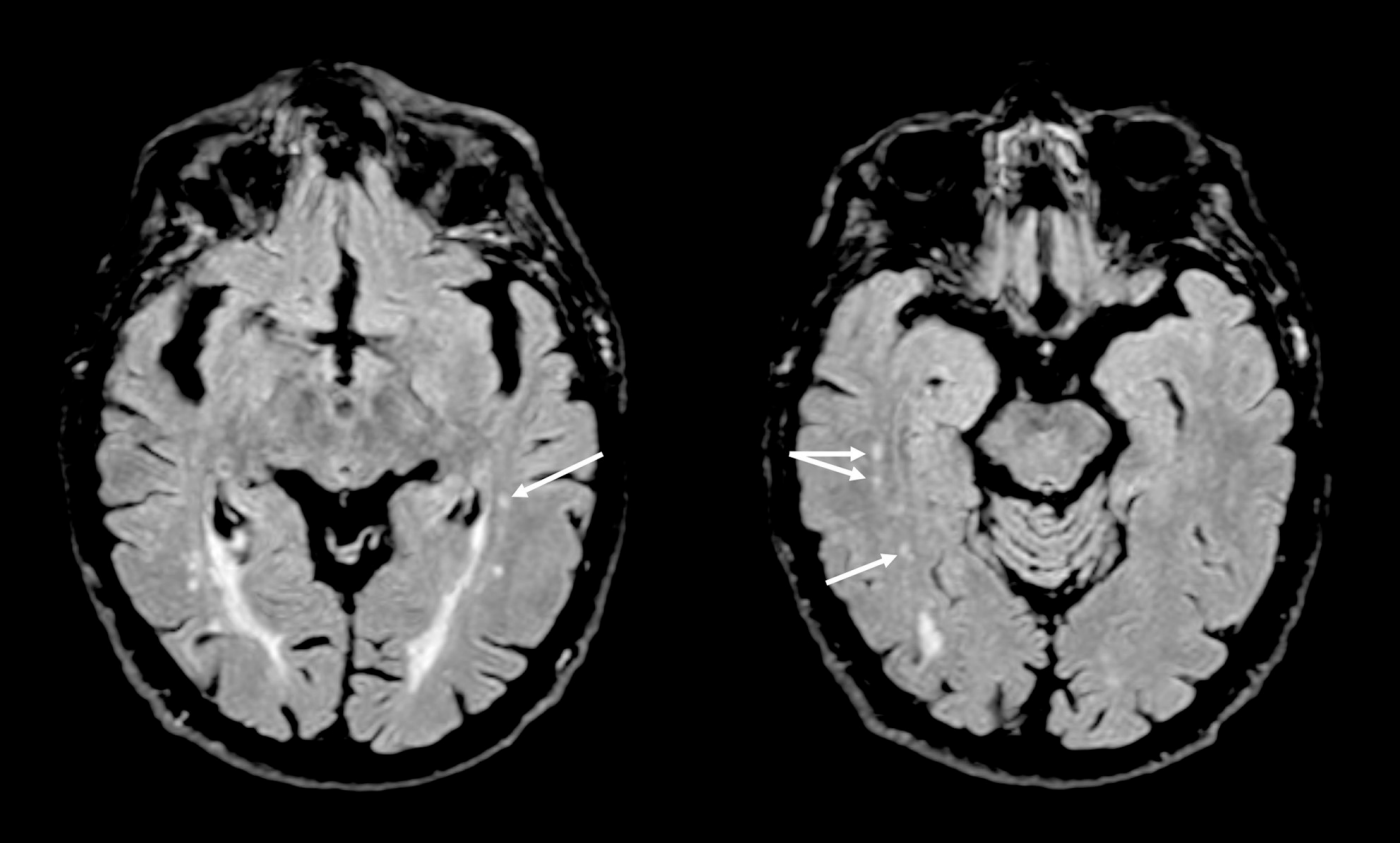
Axial T2-weighted fluid-attenuated inversion recovery (FLAIR) images showing small gliotic foci in deep white matter of both temporal lobes (arrows) in a patient with burning mouth syndrome.

A novelty of this study is the finding that patients with BMS showed a statistically significant difference in the WMCs of temporal lobe structures compared with age-matched healthy subjects (Figure 3); probably because the temporal lobe has a key role in pain and mood modulation and exhibits an abnormal activity in chronic pain (Galton et al., 2001; Ayoub et al., 2019). In addition, the presence of confluent WMCs in this brain area has been considered as the predictive of progressive medial temporal lobe atrophy, frequently detected in AD, and therefore it might be a meaningful measure of brain disease progression and an additional risk factor (Galton et al., 2001; Ayoub et al., 2019).

The multiple regression analysis shows a high prevalence of MCI in a sample of patients with BMS but it may be related not only to known predictors but also considered as an independent variable, which needs to be addressed regardless of all the other factors.

Another possible hypothesis could be that both cardiovascular and behavior predictors work in a synergic way during the time of the development of MCI. Therefore, even if every single risk factor plays a role, when considering together they could have a higher predictive value for cognitive deterioration. Indeed, the majority of these predictors remain undetected and untreated due to a delay in BMS diagnosis although we did not find any correlation with disease onset and impairments in cognitive tests. It is not possible to exclude that the diagnostic delay could have implications in cognitive performance because it is known that untreated pain may be associated with emotional distress, causing neurostructural changes that disrupt cognitive processing and deteriorate cognitive functions (Achterberg et al., 2013; Corbett et al., 2014). These changes and CI, in the time, furtherly affect the ability of patients to engage in the self-management of the pain and contributing to the loss of global functioning (Salthouse, 2012; Schiltenwolf et al., 2017).

In this study, the contribution of aging in the development of a cognitive decline in patients with BMS was not further investigated as the stratification of the sample based on different age ranges was not feasible, although it is well-known that aging may have a pivotal role in a cognitive decline (Leite-Almeida et al., 2009; Salthouse, 2012; Oosterman et al., 2013) mainly in patients affected by NCP. Indeed, the pain competes for available attentional resources of an individual (Leite-Almeida et al., 2009; Oosterman et al., 2013) and subsequently could accelerate a cognitive decline particularly in older people where attentional resources are limited, suggesting the mutual relationships between pain, age, and cognitive functions (Christensen et al., 2009; Oosterman et al., 2013). This theory is in line with the biopsychosocial frameworks of dementia proposed by Cohen-Mansfield et al. (2000), which is the net result of a broad range of predisposing, lifelong, biological, psychological, and environmental factors, some of these are fixed (not amenable to treat, e.g., education) and other tractable (may be amenable to change, e.g., mood and vascular factors) in which pain could be considered as fuel on the fire (Cerejeira et al., 2012; Livingston et al., 2020). Moreover, a growing evidence suggests that NCP and AD share some common abnormalities of the noradrenergic system in the locus coeruleus contributed to microglial dysfunction and neuroinflammation especially in the frontal cortex (Giorgi et al., 2020).

A MCI in the time might progress in dementia, therefore the early detection of high-risk population and intervention working on potentially modifiable risk factors, identified by the 2020 Lancet Commission on dementia prevention, is even more crucial in patients with BMS (Livingston et al., 2020).

In detail, the maintenance of a SBP of 130 mm Hg or less through a proper treatment with an antihypertensive drug, a decrease in alcohol use and stopping smoking, the reduction of obesity promoting later-life physical activity, the control of cholesterol and Hcy levels, and the management of neuropsychiatric symptoms are specific actions required for every patient with MCI to prevent dementia (McGrath et al., 2017; Livingston et al., 2020; Avan and Hachinski, 2021).

The control of cholesterol levels is essential in midlife because high cholesterol level appears to be a risk factor for dementia (McFarlane and Kedziora-Kornatowska, 2020), especially AD (Shobab et al., 2005) as suggested by *in vitro* studies because it is implicated in reducing the production of soluble amyloid precursor protein and the modulating α-secretease cleavage of amyloid precursor protein production (Tsatsanis et al., 2020). These actions are involved in the amyloid cascade leading to neuronal death (Shobab et al., 2005; Solfrizzi et al., 2006). In addition, a higher HDL cholesterol level might protect against the presence of vascular risk and inflammation with amyloid-β pathology in MCI (O'Brien and Wong, 2011; Button et al., 2019).

Moreover, managing psychological distress is relevant because it could predict the onset of dementia 25 years later as suggested by the Norwegian HUNT Study (Krokstad et al., 2013). In this study on 10,189 individuals with a follow-up of 10 years, they reported late-life symptoms (over 65 years) and there is an increase of dementia risk also in cognitively healthy subjects (Krokstad et al., 2013).

The MCI in patients with BMS, independently from the evolution in dementia, might increase their disability and complicate not only their own functioning but also the quality of life of their caregivers and families (Brodaty and Donkin, 2009; Mwendwa et al., 2021). For this reason, it is clear that this syndrome continues to be a challenge for clinicians particularly in the evaluation of patients that, currently, require complete multidisciplinary skills to identify high-risk patients who could develop dementia. In this context, it is essential to assess the psychological and cognitive profile over pain in all patients with BMS, and to carefully monitor patients who suddenly develop a late-life depression and sleep disturbance (over 70 years) because these symptoms could be a part of dementia prodrome (Shi et al., 2018; Ly et al., 2021).

We have speculated the use of burning fog term for all patients that self-reported symptoms of CI such as subjective concentration difficulties, forgetfulness, mental confusion, and inability to multitask, which are confirmed by a subsequent adequate neurocognitive assessment. The MCI called as burning fog in these patients could be considered as a preclinical transitional state of dementia, for which targeted interventions may be feasible because these symptoms could precede the onset of noticeable clinical signs of AD, also after 20 years, and it is never too early in the life course for dementia prevention.

Mini Mental State Examination could be the first evaluation test for the cognitive evaluation in patients with BMS as suggested in several NCP studies (Povedano et al., 2007; Rodríguez-Andreu et al., 2009; Moriarty et al., 2017; Larner, 2018). In addition, it is possible to consider as an adjuvant tool TMT because patients with BMS have shown to exhibit the worst performance compared with healthy controls in these tasks, suggesting a greater deterioration in attention and executive functions.

## Conclusions

This is the first study supporting the theory that patients with BMS show burning fog with a decrease in the global cognitive function, attention, working memory, and executive functions with a higher score of ARWMC in the temporal lobes of the brain compared with healthy subjects. It is still unclear if this CI is a primary disease manifestation or a consequence of it.

Moreover, these patients exhibit a high level of pain, the high prevalence of depression, the anxiety and sleep disturbances with a shorter sleep duration, and an impaired HRQoL compared with controls.

The CI of patients with BMS is not correlated with pain and mood disorders but with female gender, lower educational level, hypertension, and with a short sleep duration. This study confirms that BMS is a complex disease in which pain could be only the tip of the iceberg; therefore considering only the pain and mood assessment could not be satisfactory for a comprehensive evaluation of the patient.

Aging may affect the progression of WMC concurrently with a cognitive decline and subsequently it could further affect the connections in the descending modulatory system of pain, aggravating pain perception. Therefore, clinicians should consider these findings supporting a multidisciplinary assessment that may include the cognitive tests and MRI of the brain in patients with BMS at the first consultation and during the follow-up.

We recommend an individualized previous intervention in patients with BMS considering the person as a whole and therefore not to focus only on improving the pain and the psychological profile of patients with proper drugs, but also on reducing vascular risk factors, promoting correct lifestyle behaviors, and encouraging patients to keep cognitively, physically, and socially active.

These actions could have important implications in the management of BMS, improving the quality of life and delaying aging and dementia in affected patients.

Despite the limitations of the study, these results furtherly reinforce that novel multimodal drugs such as vortioxetine (Adamo et al., 2019, 2021) recently considered in the treatment of BMS could have a role not only in pain modulation and mood improvement but also in reversing brain alterations such as WMCs and enhancing a cognitive decline in patients.

Further research, on a large scale, is required to confirm our scenario and determine whether the present set of outcomes represents a specific signature of the cognitive performance in patients with BMS.

## Limitations

The study presents some limitations. Firstly, this study is a case-control study, and therefore the results of the study might be considered exploratory and should be interpreted carefully due to the nature of the study design. Secondly, it rather explores the potential associations between BMS and CI, and as a consequence it is not possible to establish a cause–effect relationship between BMS and CI for which prospective studies are needed to follow-up the patients. Thirdly, the contribution of the aging to the CI was not evaluated as the stratification of the sample based on the age was not feasible.

Finally, as this is the first study, which has evaluated CI in this population, a comprehensive set of questionnaires were used to assess all the cognitive skills and subcategories; however, it is not feasible based on this one study to advise on the use of a specific and more targeted set of tools for the clinical assessment of CI in patients with BMS.

## Data Availability Statement

The raw data supporting the conclusions of this article will be made available by the authors, without undue reservation.

## Ethics Statement

The studies involving human participants were reviewed and approved by Ethics Committee of University Of Naples Federico II - President Prof. Claudio Buccelli. The patients/participants provided their written informed consent to participate in this study.

## Author Contributions

DA, FC, and MM: conceptualization. DA, FC, EC, LU, RC, GB, and MM: methodology. LD and MA: software. MM and DA: validation. LD and MA: formal analysis. FC, EC, GM, RG, GP, LU, RC, GB, LD, MA, and MM: investigation. FC, EC, MA, and MM: resources. FC, EC, GM, RG, GP, LU, RC, GB, LD, MA, and MM: data curation. FC, DA, EC, GP, LU, and RC: writing—original draft preparation. FC, EC, GM, RG, GP, LU, RC, GB, LD, MA, and MM: writing—review and editing. DA, FC, RC, LU, and MM: visualization. DA, RC, and MM: supervision. All the authors have contributed to the work and are familiar with the primary data, each has read the final version of the manuscript, approved its content, and have agreed to have their name added to the paper.

## Conflict of Interest

The authors declare that the research was conducted in the absence of any commercial or financial relationships that could be construed as a potential conflict of interest.

## Publisher's Note

All claims expressed in this article are solely those of the authors and do not necessarily represent those of their affiliated organizations, or those of the publisher, the editors and the reviewers. Any product that may be evaluated in this article, or claim that may be made by its manufacturer, is not guaranteed or endorsed by the publisher.
