# Supplementary material for: Burning Fog: Cognitive Impairment in Burning Mouth Syndrome
Source: Front Aging Neurosci. 2021 Aug 12;13:727417. doi: 10.3389/fnagi.2021.727417 (PMC8406777; doi:10.3389/fnagi.2021.727417)
Supplement: Supplementary file 1 [file Data_Sheet_1.docx]

**Supplementary materials**

**Cognitive assessment battery**

1. **Global cognitive function**
2. **Attention**
3. **Working memory**
4. **Verbal Memory**
5. **Constructional apraxia**
6. **Executive Function**
7. **Global cognitive function**

The **Mini-Mental State Exam (MMSE)** (Folstein et al., 1975) is a brief and widely tool used for screening of cognitive impairment; it is an 11-item neurocognitive measure that assesses five areas of global cognitive functioning: orientation, attention, short-term memory, language, and visual-spatial skills (Tombaugh and McIntyre, 1992). The questions typically have been grouped into seven categories, each rationally representing a different cognitive domain or function: orientation to time (5 points); orientation to place (5 points); registration of three words (3 points); attention and calculation (5 points); recall of three words (3 points); language (8 points) and visual construction (1 point). Scores on the MMSE range from 0 to 30, with higher scores indicating better global cognitive function (Larner, 2018). The MMSE was administered for descriptive purposes only to verify that the sample under investigation was cognitively intact. The scores <24 suggest the presence of cognitive decline (23–21: mild decline; 20-11: moderate decline; and <10, severe decline) (Roselli et al., 2009). The test takes about 10 minutes to complete.

1. **Attention**

The **Trail Making Tests (TMT)** (Reitan, 1958) measures visual attention and task switching and assesses visual search, processing speed and sequencing, mental flexibility, focused attention, and executive functions (Bowie and Harvey, 2006). The most widely used version of the TMT comprises parts A and B. The two parts of the test also have different physical layouts.

Specifically, part A evaluates visual conceptual and visuomotor tracking, attention, sequencing, visual search, and motor function, whereas part B is considered a higher-level cognitive test which evaluates also motor function, attention and mental flexibility and subsequently executive function (Ashendorf et al., 2008).

In **TMT part A (TMT-A)**, the subject was assigned to draw lines sequentially connecting 26 encircled numbers as fast as possible (Zhou et al., 2017). The obtained score represents the amount of time required to complete the task. Errors do not directly contribute to the scoring and are generally not tallied. The effect of errors is realized on the total time to complete the test. The means time to complete the test in subjects with amnestic MCI is about 54.8±12.9 seconds.

The **Digit Cancellation Test (DCT)** (Spinnler et al., 1987) evaluates focused, sustained, and selective attention. We used the oDCT version Spinnler-Tognoni, 1987, which includes three different matrices composed by 13 strings of 10 digits each; the digits are in random sequence (Piacentini, 2012). The first two lines of each matrix are used as an example test. The subject has to cross or circle the target digit: the first matrix has only one target digit, the second one has two digits and the third one has three target digits, so the maximum score is 10 for the first matrix, 20 for the second one and 30 for the third one (range 0-50) (Sala et al., 1992). Score is given by the number of corrected targets crossed out within 45 seconds (time-limit) for each matrix and potential mistakes are not considered. This test takes about 5 minutes to be administered. The total score of the test in subjects with Alzheimer disease is about 23 total correct targets (Hatta et al., 2012).

1. **Working memory**

The **Corsi Block-Tapping Task (CB-TT)** (Orsini et al., 1987) is a psychological test that assesses visual short-term working memory. The examiner shows a set of 9 identical wooden blocks positioned on a board (Berch et al., 1998). The subject is required to point at the blocks in the order they are tapped. The span corresponds to the longest sequence remembered (three correct out of five). It takes at least 3-4 minutes to be administered. We have used Orsini et al. 1987 version ad scores (Orsini et al., 1987). The total score of the test in subjects with MCI is about 4.2±0.8 (Guariglia, 2007) considering the longest correct sequence carried out by the patient.

The **forward and backward** **Digit-Span task (FDS/BDS)** (Costa, 1975), evaluates short-term working memory**.** It is a simple, rapid, and reliable test to assess verbal span. In the FDS, the examiner spoke a list of two digits at a rate of one digit per second and the subject was required to repeat back the list in the same order. If he succeeded, lists of digits of increasing length were presented (Muangpaisan et al., 2010). If the subject failed on a list, a second list of the same length was given and if the subject was successful, a list one digit longer was then given as before. If, on the contrary, the subject failed also the second list the test was stopped. The span was established as the length of the last list recalled correctly. The same procedure was followed for the BDS with the difference that, in this case, subjects were requested to reproduce the sequence of digits in the reversed order. This test takes at least 4 minutes to be performed. We used the clinical protocol of digit-span forward and backward of Monaco et al. (Monaco et al., 2013). The total score for FDS in patients with MCI is about 10.6±1.8 while for BDS is 3.9 ± 1.3 considering the longest correct sequence carried out by the patient (Muangpaisan et al., 2010).

1. **Verbal Memory**

The **Rey Auditory Verbal Learning Test (RAVLT)** (Rey, 1941) evaluates episodic memory (the ability to recall and mentally reexperience specific episodes from one's personal past) and the verbal learning ability (Vuoksimaa et al., 2020). The RAVLT evaluates verbal memory disfunctions and it is able to control memory function over time. During the test the examiner reads a list of 15 words with the rhythm of one word per second; every time the examiner repeats the list (five times), the participant has to repeat the words he remembers in random order. The total of remembered words in all the five repetitions is the score of immediate recalling (range 0-75). After a 15 min delay, during which the participant does not carry out verbal activities, he is asked to again recall the words from the list. This delayed recall has a different score (range 0-15). The RAVLT score in individuals with amnestic MCI (Knight et al., 2007), according to the Alzheimer’s Disease Neuroimaging Initiative criteria, is about 30.64±8.97 while for the RAVLT delayed is about 2.81±3.26. considering the number of words correctly repeated (Van Der Elst et al., 2005).

1. **Constructional apraxia**

The **Copying Geometric Drawings test (CGD)** (Spinnler et al., 1987) assesses the constructional apraxia in order to measure impairments in processing spatial forms (Ward et al., 2015); it evaluates the ability of patient to copy geometric figures shown in 7 tables. There is also a preliminary table with two shapes that will not be considered in the score, in table 1 there is a square, in table 2 a rhombus, in table 3 a one line complex shape, in table 4 a cube, in table 5 there are a little square, a circle and an isosceles triangle placed side by side, in table 6 there are an equilateral triangle, a divided rhombus and a little isosceles triangle placed side by side, in table 7 a complex figure composed of 8 triangles (Trojano and Grossi, 1998). The score is based on good or poor quality of the drawings (2 points for a perfect copy, 1 point if the copy is partially flawed, 0 point if the copy is unrecognizable or there is closing-in). The total score range is 0-14. This test takes at least 10 minutes to be administered. We used adjusted scores following normative value of Spinnler e Tognoni (Spinnler et al., 1987) considering a cut-off total score for MCI of 8 points (Gainotti and Trojano, 2018).

1. **Executive Function**

**The TMT part B (TMT-B)** (Reitan, 1958) measures mental flexibility, motor function, attention with a focus on set-shifting. Participants are required to connect circled numbers and letters in alternating numeric and alphabetic sequence (i.e., 1, A, 2, B, etc.) starting from 1 and ending with 13. If an error is made, the test administrator corrects the examinee before advancing to the next circle. Response time (in seconds) is recorded, with a longer response time representing poorer set-shifting performance (Bowie and Harvey, 2006). The total score corresponds with the time taken to complete the subtest; in subject with MCI the time to complete the test is about 164.3±70.2 sec (Ashendorf et al., 2008).

**TMT Delta (TMT-B-TMT-A)** is the difference between time taken to complete TMT parts B and A, and it is used to control information processing speed and motor function; it is recognized as a direct measure of executive functioning (Zhou et al., 2017). TMT Delta is measured in seconds with a shorter time reflecting better perform.

The **Frontal Assessment Battery (FAB)** (Dubois et al., 2000) consists of 6 subtests exploring different functions related to the frontal lobes (Hurtado-Pomares et al., 2018). The 6 subtests of the FAB explore the following: (1) conceptualization and abstract reasoning (similarities test); (2) mental flexibility (verbal fluency test); (3) motor programming and executive control of action (Luria motor sequences); (4) resistance to interference (conflicting instructions); (5) inhibitory control (go–no go test); and (6) environmental autonomy (prehension behavior). Each subtest is scored from 3 (better score) to 0, for a maximum score of 18. We used adjusted scores following normative value of Apollonio et al. 2005 (Appollonio et al., 2005). The total FAB score in subjects with MCI is about 13.1 ± 2.1; higher scores indicating a better performance (Goh et al., 2019). This test requires approximately 10 minutes to complete and is easy to administer.

**References**

Appollonio, I., Leone, M., Isella, V., Piamarta, F., Consoli, T., Villa, M. L., et al. (2005). The Frontal Assessment Battery (FAB): normative values in an Italian population sample. *Neurol Sci* 26, 108–116. doi:10.1007/s10072-005-0443-4.

Ashendorf, L., Jefferson, A., Oconnor, M., Chaisson, C., Green, R., and Stern, R. (2008). Trail Making Test errors in normal aging, mild cognitive impairment, and dementia. *Archives of Clinical Neuropsychology*, S0887617707002247. doi:10.1016/j.acn.2007.11.005.

Berch, D. B., Krikorian, R., and Huha, E. M. (1998). The Corsi Block-Tapping Task: Methodological and Theoretical Considerations. *Brain and Cognition* 38, 317–338. doi:10.1006/brcg.1998.1039.

Bowie, C. R., and Harvey, P. D. (2006). Administration and interpretation of the Trail Making Test. *Nat Protoc* 1, 2277–2281. doi:10.1038/nprot.2006.390.

Costa, L. D. (1975). The relation of visuospatial dysfunction to digit span performance in patients with cerebral lesions. *Cortex* 11, 31–36. doi:10.1016/s0010-9452(75)80018-9.

Dubois, B., Slachevsky, A., Litvan, I., and Pillon, B. (2000). The FAB: a Frontal Assessment Battery at bedside. *Neurology* 55, 1621–1626. doi:10.1212/wnl.55.11.1621.

Folstein, M. F., Folstein, S. E., and McHugh, P. R. (1975). “Mini-mental state”. A practical method for grading the cognitive state of patients for the clinician. *J Psychiatr Res* 12, 189–198. doi:10.1016/0022-3956(75)90026-6.

Gainotti, G., and Trojano, L. (2018). “Constructional apraxia,” in *Handbook of Clinical Neurology* (Elsevier), 331–348. doi:10.1016/B978-0-444-63622-5.00016-4.

Goh, W. Y., Chan, D., Ali, N. B., Chew, A. P., Chuo, A., Chan, M., et al. (2019). Frontal Assessment Battery in Early Cognitive Impairment: Psychometric Property and Factor Structure. *J Nutr Health Aging* 23, 966–972. doi:10.1007/s12603-019-1248-0.

Guariglia, C. C. (2007). Spatial working memory in Alzheimer’s disease: A study using the Corsi block-tapping test. *Dement. neuropsychol.* 1, 392–395. doi:10.1590/S1980-57642008DN10400011.

Hatta, T., Yoshizaki, K., Ito, Y., Mase, M., and Kabasawa, H. (2012). RELIABILITY AND VALIDITY OF THE DIGIT CANCELLATION TEST, A BRIEF SCREEN OF ATTENTION. *An International Journal of Psychological Sciences* 55, 246–256. doi:10.2117/psysoc.2012.246.

Hurtado-Pomares, M., Carmen Terol-Cantero, M., Sánchez-Pérez, A., Peral-Gómez, P., Valera-Gran, D., and Navarrete-Muñoz, E. M. (2018). The frontal assessment battery in clinical practice: a systematic review: Clinical practice of the FAB. *Int J Geriatr Psychiatry* 33, 237–251. doi:10.1002/gps.4751.

Knight, R., Mcmahon, J., Skeaff, C., and Green, T. (2007). Reliable Change Index scores for persons over the age of 65 tested on alternate forms of the Rey AVLT. *Archives of Clinical Neuropsychology* 22, 513–518. doi:10.1016/j.acn.2007.03.005.

Larner, A. J. (2018). Mini-Mental State Examination: diagnostic test accuracy study in primary care referrals. *Neurodegenerative Disease Management* 8, 301–305. doi:10.2217/nmt-2018-0018.

Monaco, M., Costa, A., Caltagirone, C., and Carlesimo, G. A. (2013). Forward and backward span for verbal and visuo-spatial data: standardization and normative data from an Italian adult population. *Neurol Sci* 34, 749–754. doi:10.1007/s10072-012-1130-x.

Muangpaisan, W., Intalapaporn, S., and Assantachai, P. (2010). Digit Span and Verbal Fluency Tests in Patients with Mild Cognitive Impairment and Normal Subjects in Thai-Community. 93, 7.

Orsini, A., Grossi, D., Capitani, E., Laiacona, M., Papagno, C., and Vallar, G. (1987). Verbal and spatial immediate memory span: Normative data from 1355 adults and 1112 children. *Ital J Neuro Sci* 8, 537–548. doi:10.1007/BF02333660.

Piacentini, S. (2012). “Valutazione neuropsicologica,” in *Guida alla valutazione medico-legale del danno neurologico*, eds. A. Sghirlanzoni and U. Genovese (Milano: Springer Milan), 201–226. doi:10.1007/978-88-470-2074-0_20.

Reitan, R. M. (1958). Validity of the Trail Making Test as an Indicator of Organic Brain Damage. *Percept Mot Skills* 8, 271–276. doi:10.2466/pms.1958.8.3.271.

Rey, A. (1941). L’examen psychologique dans les cas d’encéphalopathie traumatique. (Les problems.). [The psychological examination in cases of traumatic encepholopathy. Problems.]. *Archives de Psychologie* 28, 215–285.

Roselli, F., Tartaglione, B., Federico, F., Lepore, V., Defazio, G., and Livrea, P. (2009). Rate of MMSE score change in Alzheimer’s disease: Influence of education and vascular risk factors. *Clinical Neurology and Neurosurgery* 111, 327–330. doi:10.1016/j.clineuro.2008.10.006.

Sala, S. D., Laiacona, M., Spinnler, H., and Ubezio, C. (1992). A cancellation test: its reliability in assessing attentional deficits in Alzheimer’s disease. *Psychol. Med.* 22, 885–901. doi:10.1017/S0033291700038460.

Spinnler, H., Tognoni, G., and Gruppo italiano per lo studio neuropsicologico dell’invecchiamento (1987). *Standardizzazione e taratura italiana di test neuropsicologic*. Milano: Masson Italia Periodici.

Tombaugh, T. N., and McIntyre, N. J. (1992). The Mini-Mental State Examination: A Comprehensive Review. *Journal of the American Geriatrics Society* 40, 922–935. doi:10.1111/j.1532-5415.1992.tb01992.x.

Trojano, L., and Grossi, D. (1998). ‘Pure’ Constructional Apraxia—A Cognitive Analysis of a Single Case. *Behavioural Neurology* 11, 43–49. doi:10.1155/1998/614728.

Van Der Elst, W., Van Boxtel, M. P. J., Van Breukelen, G. J. P., and Jolles, J. (2005). Rey’s verbal learning test: Normative data for 1855 healthy participants aged 24–81 years and the influence of age, sex, education, and mode of presentation. *J Int Neuropsychol Soc* 11, 290–302. doi:10.1017/S1355617705050344.

Vuoksimaa, E., McEvoy, L. K., Holland, D., Franz, C. E., Kremen, W. S., and Alzheimer’s Disease Neuroimaging Initiative (2020). Modifying the minimum criteria for diagnosing amnestic MCI to improve prediction of brain atrophy and progression to Alzheimer’s disease. *Brain Imaging Behav* 14, 787–796. doi:10.1007/s11682-018-0019-6.

Ward, M., Cecato, J. F., Aprahamian, I., and Martinelli, J. E. (2015). Assessment for apraxia in Mild Cognitive Impairment and Alzheimer’s disease. *Dement. neuropsychol.* 9, 71–75. doi:10.1590/S1980-57642015DN91000011.

Zhou, H., Sabbagh, M., Wyman, R., Liebsack, C., Kunik, M. E., and Najafi, B. (2017). Instrumented Trail-Making Task to Differentiate Persons with No Cognitive Impairment, Amnestic Mild Cognitive Impairment, and Alzheimer Disease: A Proof of Concept Study. *Gerontology* 63, 189–200. doi:10.1159/000452309.
